# Next generation sequencing gives an insight into the characteristics of highly selected breeds versus non-breed horses in the course of domestication

**DOI:** 10.1186/1471-2164-15-562

**Published:** 2014-07-04

**Authors:** Julia Metzger, Raul Tonda, Sergi Beltran, Lídia Águeda, Marta Gut, Ottmar Distl

**Affiliations:** Institute for Animal Breeding and Genetics, University of Veterinary Medicine Hannover, Bünteweg 17p, 30559 Hannover, Germany; Centro Nacional de Análisis Genómico, Parc Científic de Barcelona, Torre I Baldiri Reixac, 4, Barcelona, 08028 Spain

## Abstract

**Background:**

Domestication has shaped the horse and lead to a group of many different types. Some have been under strong human selection while others developed in close relationship with nature. The aim of our study was to perform next generation sequencing of breed and non-breed horses to provide an insight into genetic influences on selective forces.

**Results:**

Whole genome sequencing of five horses of four different populations revealed 10,193,421 single nucleotide polymorphisms (SNPs) and 1,361,948 insertion/deletion polymorphisms (indels). In comparison to horse variant databases and previous reports, we were able to identify 3,394,883 novel SNPs and 868,525 novel indels. We analyzed the distribution of individual variants and found significant enrichment of private mutations in coding regions of genes involved in primary metabolic processes, anatomical structures, morphogenesis and cellular components in non-breed horses and in contrast to that private mutations in genes affecting cell communication, lipid metabolic process, neurological system process, muscle contraction, ion transport, developmental processes of the nervous system and ectoderm in breed horses.

**Conclusions:**

Our next generation sequencing data constitute an important first step for the characterization of non-breed in comparison to breed horses and provide a large number of novel variants for future analyses. Functional annotations suggest specific variants that could play a role for the characterization of breed or non-breed horses.

**Electronic supplementary material:**

The online version of this article (doi:10.1186/1471-2164-15-562) contains supplementary material, which is available to authorized users.

## Background

The process of domestication has shaped the modern horse population and lead to an immense group of different types of breeds [1, 2]. Various environmental as well as artificial factors affected the population structure and lead to the formation of more than 400 horse breeds today [2, 3]. In order to create horses with a characteristic uniform appearance and function, some breeds have especially been under strong directional selection to a special breeding goal while other populations still underlie a greater natural selection and have kept their original properties to survive under harsh environment [2, 4]. Despite human influences, the Duelmener horse as well as the Sorraia developed under quite natural circumstances as they are generally kept under free range conditions without specific human care for health. They show typical primitive markings and a robust constitution for the survival under harsh conditions [5–7]. Those horses which are less subjected to a breeding goal but to the preservation of this specific population can be grouped as non-breed horses [2]. These non-breeds developmentally lie in-between the highly selected modern horse breeds and the Przewalski population which falls outside of the monophyletic group of domestic horses and represents the last survivor of wild horses [8]. In contrast to that the Hanoverian as well as the Arabian, one of the oldest recognized domestic breeds, have been subject to close breeding and intense human selection for their high ability in function and performance [9, 10]. A comparative whole genome sequence analysis with the genomes of five modern domestic horses identified private loci selected for modern horses and suggested a continuous selection on the immune system and olfaction throughout horse evolution. Other genomic regions showed low levels of genetic variation compared to the Przewalski horse and were suggested to be potentially selected early during domestication [8]. Further specific modern domestic horse variants have been investigated by next generation sequencing of a Quarter horse mare that provided 2.8 million novel SNPs, 193 thousand indels and 282 CNVs and revealed an enrichment of biological processes involved in sensory perception, signal transduction, immunity and defense pathways [11]. The aim of this study was to investigate the influence of human selection on domestic horses by comparative analysis of non-breed to breed horses. Next generation sequencing was performed to characterize five horses of two different breeds and two different non-breeds representing the highly diverse horse population by the detection of novel genetic variations. The results show that the process of domestication as well as further artificial selection for specific breeding aims has influenced the development of specific biological processes in horse breeds.

## Results

### Sequence analysis and variant detection

Whole genome sequencing was performed in two Hanoverians and one Arabian representing breed horses and one Duelmener and Sorraia representing non-breed horses using the Illumina HiSeq2000 (Illumina, San Diego, CA). Each horse was run on one lane except one of the Hanoverians (Hanoverian 1) which was run on two lanes due to its importance for the Hanoverian breed. After passing filters, reads were trimmed and aligned to the reference horse genome derived from the Thoroughbred mare Twilight and its half-brother Bravo (EquCab2.70, Additional file 1A) [12]. The alignment resulted in a mean coverage of 14.02X for the Duelmener, 12.21X for the Sorraia, 13.37X for the Arabian, 10.97X for Hanoverian 2 and a higher mean coverage of 25.38X for Hanoverian 1 due to its run on two lanes (Additional file 1B). In total 67.38% of the reference genome showed at least 10X sequence coverage in the Duelmener, 52.87% in the Sorraia, 62.54% in the Arabian and 40.86% in Hanoverian 2. The two lane run for Hanoverian 1 increased the average number of reads and resulted in a 10X sequence coverage in 96.65 of the reference genome. The aligned sequence was further processed for variant detection and revealed 5,391,494 SNPs in the Duelmener, 5,075,637 in the Sorraia, 5,156,659 in the Arabian, 5,264,058 in Hanoverian 1 and 5,032,162 in Hanoverian 2. On the whole, 1,712,330 SNPs were shared by all five horses (Additional file 1C). All horses had 551,444 indels in common, while the individual number of indels was at 889,106-935,333. In total our analysis revealed 10,193,421 SNPs and 1,361,948 indels. Heterozygosity was 0.317 SNPs/site in the Duelmener, 0.300 SNPs/site in the Arabian, 0.342 SNPs/site in Hanoverian 1, 0.309 SNPs/site in Hanoverian 2 and 0.244 SNPs/site in the Sorraia. Considering the distribution of variants identified on the individual chromosomes of non-breed and breed horses, these groups showed an almost identical number of variants per chromosome (Additional file 2). The highest number of variants could be detected on chromosome (ECA) 1. Nevertheless, with regard to the chromosomal enrichment of detected variants accounted by dividing the number of variants by the length of the chromosomes (bp), we could show that especially ECA12 and ECA20 were over the threshold of the whole genome enrichment (Additional file 3). On the whole, 19 chromosomes of the analyzed 32 chromosomes and the mitochondrial DNA exceeded the threshold of the average number of variants per base pair of the whole genome.

### Comparison with Equine SNP50 genotyping beadchip and public databases

**Figure 1:**
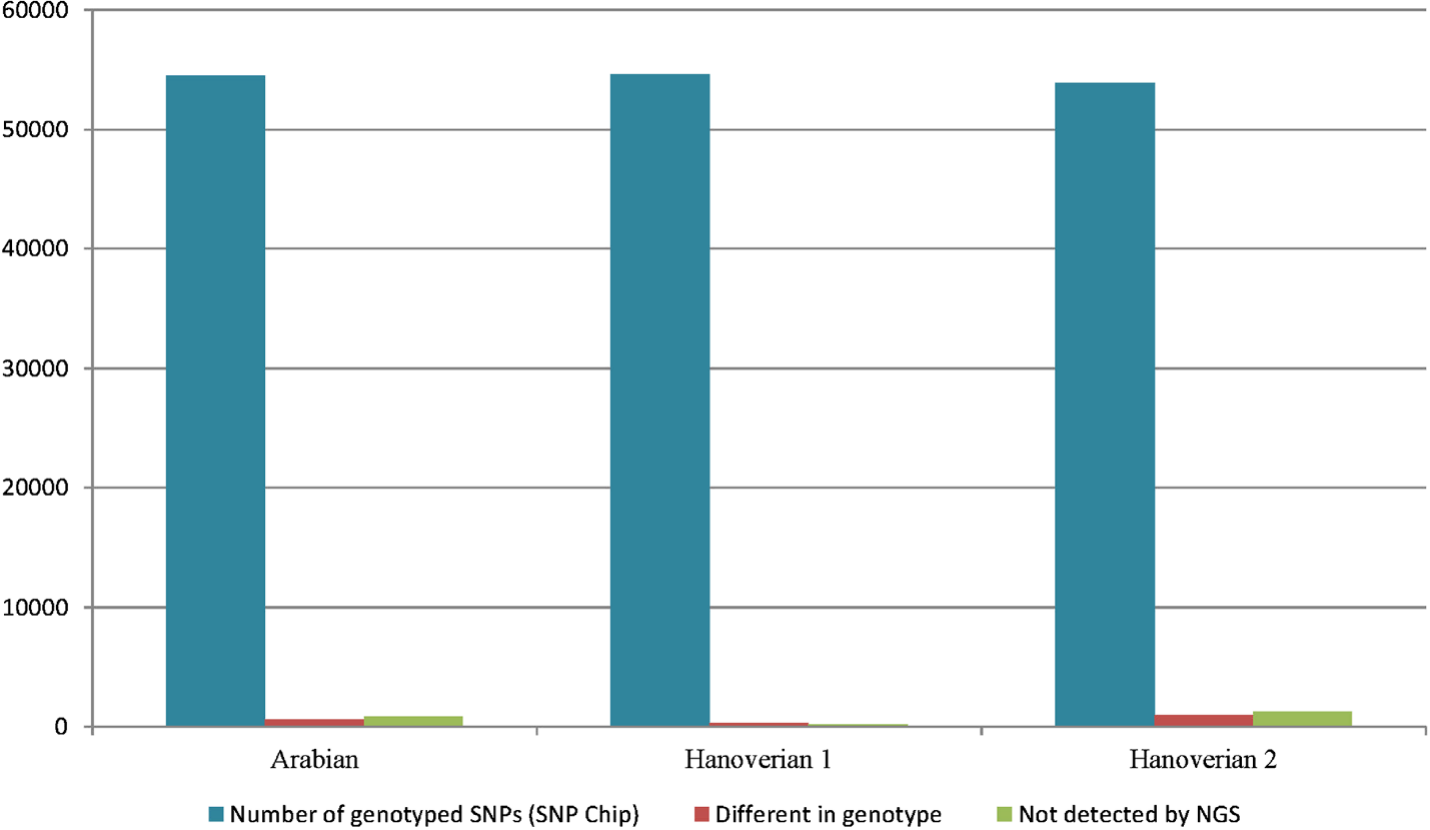
**Comparison between SNPChip and NGS data.** All three horses genotyped by SNPchip and NGS show a low frequency of SNPs different in genotype or not detected by NGS.

Comparative analyses of the detected SNPs by next generation sequencing with Equine SNP50 genotyping beadchip data in the Arabian and the two Hanoverian showed that the variant detection was reliable for these data. In total, 95.92% of the SNPs from beadchip analysis could be confirmed by next generation sequencing for the Arabian and 97.42% for the Hanoverian (Hanoverian 2) run on one lane (Figure 1). The Hanoverian which was run on two lanes (Hanoverian 1) even showed a concordance with beadchip data of 99.13%. Only 0.51% of the SNPs were different in genotype and 0.36% was not detected by next generation sequencing.

**Figure 2:**
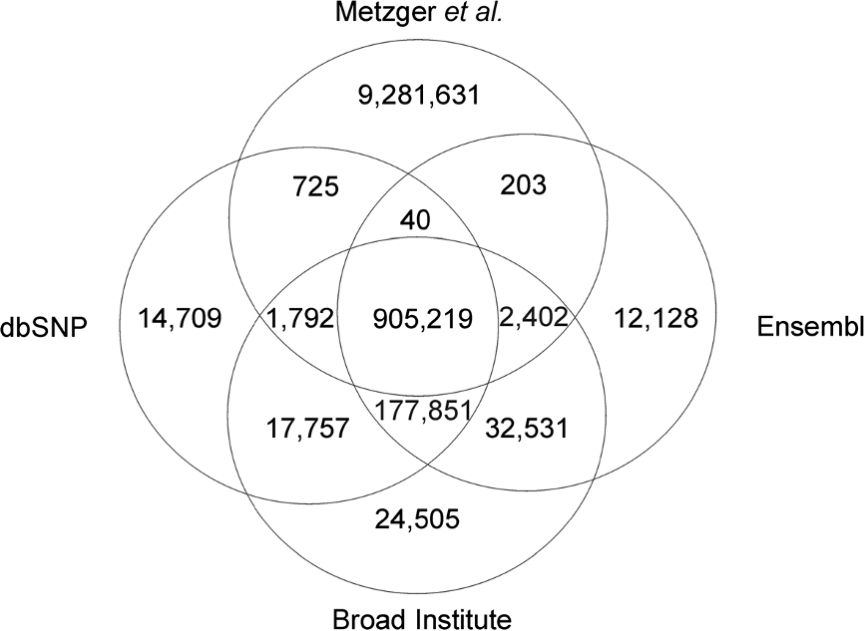
**Comparison of SNP data from present study with known SNPs in various databases.** The number of common SNPs with dbSNP, Ensembl and Broad Institute databases and the number of novel SNPs from present analysis is shown.

**Figure 3:**
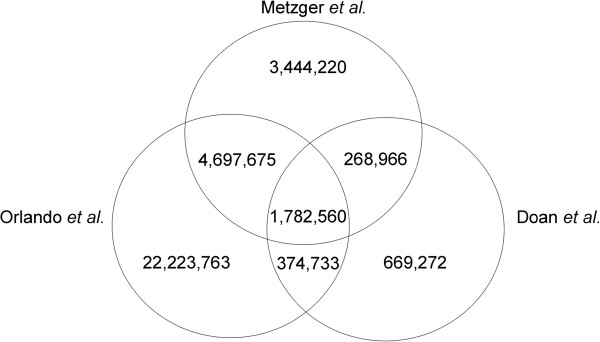
**Comparison of SNP data from present study with known SNPs in previous NGS analyses.** The number of common SNPs with Orlando *et al.* and Doan *et al.* and the number of novel SNPs from present analysis is shown.

**Figure 4:**
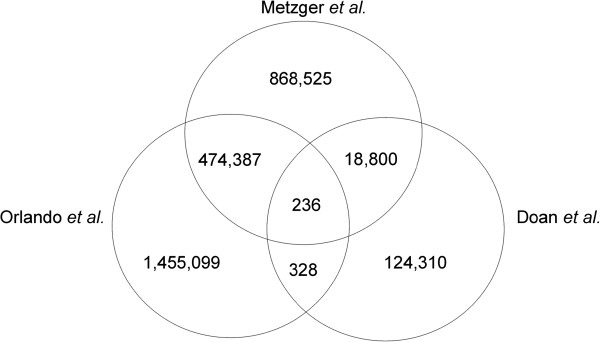
**Comparison of indel data from present study with known SNPs in previous NGS analyses.** The number of common indels with Orlando *et al.* and Doan *et al.* and the number of novel SNPs from present analysis is shown.

Further comparison was performed with SNP and indel data from open access data bases with the present variant detection results. A total of 907,776 SNPs of our analysis showed an overlap with dbSNP (dbSNP, ftp://ftp.ncbi.nih.gov/snp/organisms/horse_9796/chr_rpts/), 907,864 SNPs with Ensembl (ftp://ftp.ensembl.org/pub/release-73/variation/gvf/equus_caballus/ Equus_caballus.gvf.gz) and 910,822 SNPs with Broad Institute (http://www.broadinstitute.org/ftp/distribution/horse_snp_release/v2/) data, while 9,281,631 SNPs could not be retrieved from these databases (Figure 2). In comparison with NGS data from previous studies in a Quarter horse with a minimum read depth coverage of 10X [11] and in five domestic horses (Arabian, Icelandic, Norwegian fjord, Standardbred, Thoroughbred, 7.9-21.1X), one Przewalski (9.6X) and one donkey (16X) [8], we found 1,782,560 shared SNPs, 268,966 SNPs known from the Quarter horse data and 4,697,675 exclusively overlapping with SNPs derived from the five domestic horses, Przewalski or donkey (Figure 3). In total 3,444,220 SNPs could not be found in these published whole genome sequence data. With regard to all previously published databases and sequence analyses we detected 3,394,883 novel SNPs. Comparative analyses of our indel data with previous studies revealed 236 indels shared in all projects, 18,800 indels known from the Quarter horse and 474,387 indels overlapped the study with the five domestic horses, a Przewalski and a donkey [8]. In total 868,525 indels were exclusive in our data (Figure 4). With regard to the mutation type a huge number of 15,012 novel non-synonymous SNPs were predicted as well as SNPs affecting splice sites, start and stop codons (Table 1, Additional file 4). The novel indels in our analysis were proposed to have different effects like codon deletions, codon insertions and a large number of 4,916 frame shifts as well as four exon deletions could be found.

**Table 1:** **Comparison of exonic SNPs and indels from current analysis with known variants from different databases**

SNPEff terms by type	Total	Common SNPs with dbSNP	Common SNPs with ensembl	Common SNPs with broad	Common variants with Orlando ***et al.***[ [8]]	Common variants with Doan ***et al.***[ [11]]	Novel variants
**SNPs**							
Exon	8056	679	678	693	4650	1974	2920
Non-synonymous coding	43043	3014	3002	3026	25200	12759	15007
Non-synonymous start	10	1	1	1	3	3	5
Start lost	48	4	4	4	30	19	15
Stop gained	383	16	16	16	185	81	182
Stop lost	29	6	6	6	20	5	8
Synonymous coding	52965	4367	4355	4368	34360	16144	15962
Synonymous start	1	0	0	0	0	0	1
Synonymous stop	27	0	0	0	15	11	7
**Indels**							
Codon change plus codon deletion	133	-	-	-	31	0	102
Codon change plus codon insertion	248	-	-	-	62	0	186
Codon deletion	201	-	-	-	55	1	145
Codon insertion	178	-	-	-	39	2	137
Exon	828	-	-	-	284	26	518
Exon deleted	4	-	-	-	0	0	4
Frameshift	7360	-	-	-	1903	541	4916
Start lost	7	-	-	-	2	0	5
Stop gained	25	-	-	-	6	0	19

The total number of SNPs and indels per SNPEff term detected in five horses and their concordance with dbSNP, Broad Institute and Ensembl data as well as data published by Orlando *et al.* [8] and Doan *et al.* [11] are shown.

### Functional annotation and characterization

**Figure 5:**
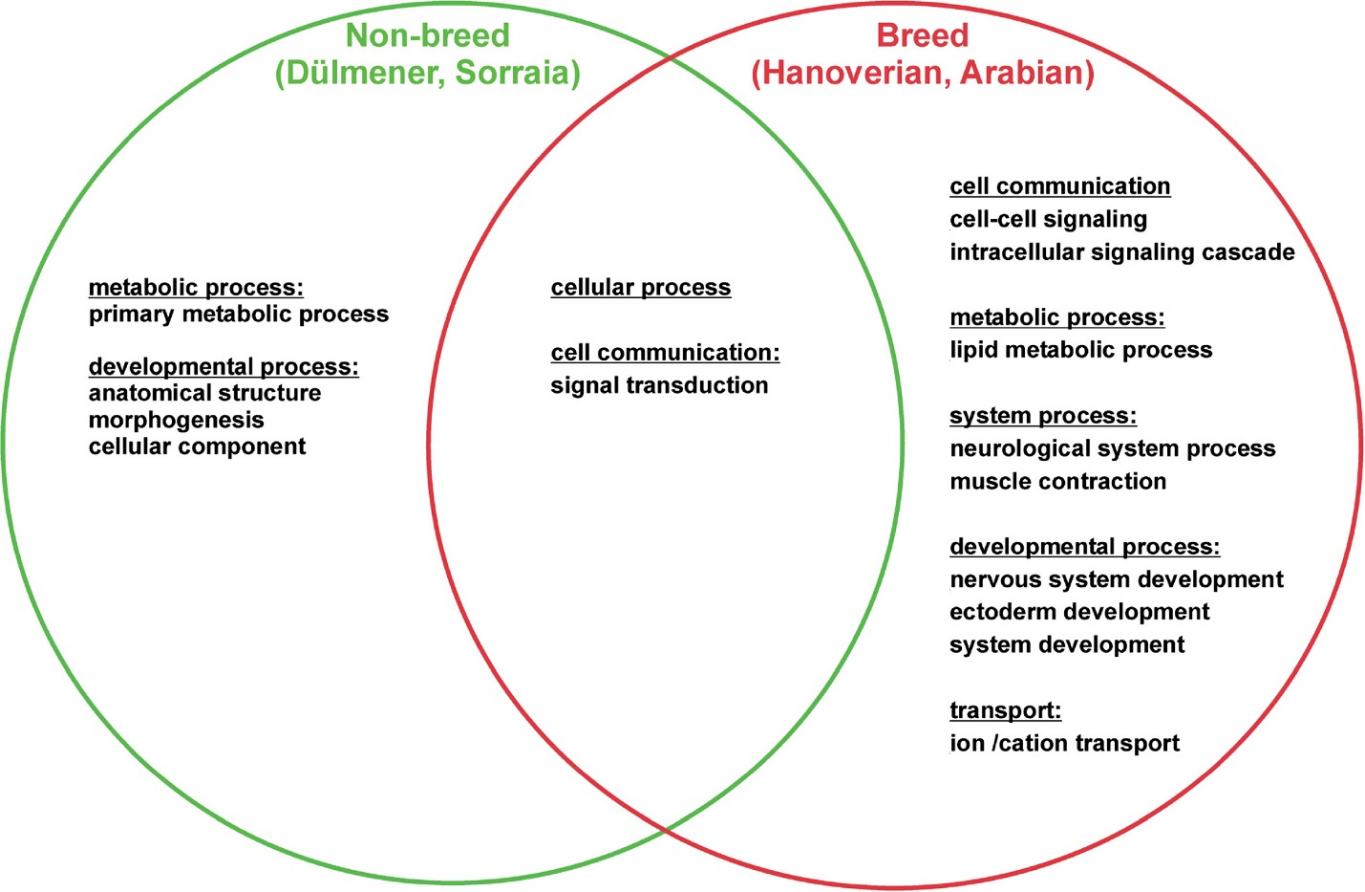
**Enrichment analysis of private variants affecting coding regions of breed and non-breed horses.** Significantly enriched gene ontology (GO) terms common or specific for breed and non-breed horses are shown.

We investigated the variants detected in our analysis for their effects on basis of the variant annotation and effect prediction tool SNPEff [13] and their distribution across breed and non-breed horses. Most of the variants revealed one or two predicted SNPEff-effects. Regarding the private SNPs that could only be found in one of the compared horses or groups, non-breed horses showed a larger number of effects by private SNPs in intergenic as well as genic regions in comparison with breed horses (Table 2). A total of 810,687 effects could be detected affected by private SNPs in the Duelmener and 824,072 effects in the Sorraia. The Arabians showed 737,324 effects while the Hanoverian horses revealed an even lower number of effects (316,464) as a result of a reduced number of private SNPs due to the analysis of two horses. With regard to specific effects, 9,507 non-synonymous SNPs were predicted to be shared by all horses while 3,053 private SNPs in the Duelmener, 2,367 private SNPs in the Sorraia, 2,191 private SNPs in the Arabian and 878 private SNPs in the Hanoverian horses. Nevertheless, functional classification analysis of the distribution of genes affected by private non-synonymous SNPs did not reveal any significant differences in these groups (Additional file 5). Other effects like splice site donors, splice site acceptors, start and stop codon changes were also predicted for shared and private variants. We further investigated the loss of the stop codons possibly affected by private SNPs and identified the genes *ATP13A4* (*probable cation-transporting ATPase 13A4*) in the Duelmener, *ENSECAG00000000628* (*TRBV6-4, T cell receptor beta variable 6–4*) and *ENSECAG00000007186* (*OR2A2, olfactory receptor, family 2, subfamily A, member 2*) in the Sorraia and *RTDR1* (*Rhabdoid tumor deletion region gene 1*) and ENSECAG00000008382 in the Hanoverians but no loss of stop codons in the Arabian (Additional file 6). All private losses of stop mutations were heterozygous for the respective individuals. Analyses of the indels revealed 629,686 effects predicted for shared variants in the five horses. Especially codon changes, splice site modifications, frameshift mutations as well as one exon deletion became apparent in the detection of private indels (Additional file 6). The heterozygous 21 bp deletion detected exclusively in the Duelmener was predicted to affect the gene *CNDP2* (*dipeptidase 2, metallopeptidase M20 family*) (Additional file 7). Further investigation of the predicted codon changes due to private indels revealed an increased occurrence of genes involved in immune system processes in breed horses (22.6%) in comparison to non-breed horses (6.7%, Additional file 7 and 8). The mean heterozygosity in immunity related regions could be shown to be considerably high in breed horses (0.30-0.33) in contrast to the Duelmener (0.24) and Sorraia (0.09). We performed an enrichment analysis for coding and regulative regions affected by SNPs for breed and non-breed horses. An analysis of coding regions in non-breed horses revealed an enrichment of genes involved in primary metabolic processes as well as genes involved in anatomical structures, morphogenesis and cellular components (Figure 5, Additional file 8). Breed horses showed an enrichment of genes in coding regions involved in cell communication, lipid metabolic process, neurological system process, muscle contraction, ion transport and developmental processes of the nervous system and ectoderm. Regulative regions with private non-breed SNPs were enriched with genes affecting proteolysis and fatty acid metabolic processes, while breed horses showed an enrichment of genes in system processes, exocytosis, developmental processes, cell communication, transport and sensory perception of sound (Additional file 9).

**Table 2:** **Number of effects by private and shared SNPs detected by next generation sequencing of five horses**

SNPEff terms by type	Shared (all 5 horses)	Duelmener	Sorraia	Arabian	Hanoverian (two horses)
Downstream	89229	43289	34418	31295	13056
Exon	1728	694	494	48	192
Intergenic	1313808	711015	549696	487818	207619
Intron	451550	256400	199575	180419	79926
Non-synonymous coding	9507	3053	2367	2191	878
Non-synonymous start	7	0	0	0	0
Splice site acceptor	159	15	10	11	3
Splice site donor	260	23	19	16	7
Start gained	159	57	43	17	10
Start lost	15	0	0	0	0
Stop gained	48	36	28	29	16
Stop lost	8	1	2	0	2
Synonymous coding	8138	3883	3030	2686	1198
Synonymous start	1	0	0	0	0
Synonymous stop	1	4	4	1	1
Upstream	113982	45203	36447	32191	13289
3‘UTR	1240	607	488	437	191
5‘UTR	1682	243	178	165	76
total	1584011	810687	824072	737324	316464

The presented results are classified by SNPEff terms for each breed.

Further investigation of known variants revealed a confident detection of the basic coat color black/chestnut and bay phenotypes (Table 3) [14]. Analysis of coat color genotypes associated with a disease did not reveal any mutations. Further search for disease traits confirmed that the Arabian was heterozygous at the *TOE1*:g.2171G > A SNP which is associated with genetic carriers for cerebellar abiotrophy [15] (Table 4). Performance trait analysis further characterized the different horse breeds. The Duelmener, Sorraia and Arabian showed a T/T genotype for the polymorphism BIEC2-808543 which was proposed to affect the *ligand dependent nuclear receptor corepressor-like (LCORL)* as the main regulator for body size in horses [16–18]. The larger sized Hanoverians had a heterozygous C/T genotype (Table 5). Five SNPs affecting racing performance were also investigated and suggested the Arabian and Hanoverians to be the horses with good stamina in long distances. Especially one Hanoverian was heterozygous for all racing performance traits. The Duelmener showed a heterozygous genotype for the g.66493737C/T mutation in the *myostatin gene (MSTN)* which is associated with middle distance racing ability and two further mutations associated with good racing performance [19]. In contrast to the Duelmener, the Sorraia showed the C/C genotype in the *MSTN* gene which can be found in fast horses in short distances.

**Table 3:** **Investigation of known variants affecting coat colors and color phenotypes associated with disease traits**

Phenotype	ECA	Position	Gene	Associated genotype	Genotype Duelmener	Genotype Arabian	Genotype Sorraia	Genotype Hanoverian 1	Genotype Hanoverian 2	References
Chestnut/black	3	36,259,552	*MC1R*	C/T substitution: Ser/Phe	C/C	T/T	C/C	T/T	C/T	[14]
Chestnut	3	36,259,554	*MC1R*	G/A substitution: Asp/Asn	G/G	G/G	G/G	G/G	G/G	[20]
Bay/black	22	25,168,579	*ASIP*	11 bp deletion: frameshift	ref/ref	ref/ins	ref/ref	ref/ref	ref/ins	[14]
Grey	25	6,575,277	*STX17*	4.6 kb duplication	Not detected	Not detected	Not detected	Not detected	Not detected	[21]
Cream	21	30,666,626	*SLC45A2*	11 bp deletion: frameshift	Not detected	Not detected	Not detected	Not detected	Not detected	[22]
Champagne	14	26,701,092	*SLC36A1*	C/G substitution: Thr/Arg	G/G	G/G	G/G	G/G	G/G	[23]
Splashed white	16	20,117,302	*MITF*	11bp deletion-insertion	Not detected	Not detected	Not detected	Not detected	Not detected	[24, 25]
Splashed white	16	20,105,348	*MITF*	DelGTGTC	Not detected	Not detected	Not detected	Not detected	Not detected	[24, 25]
Splashed white	6	11,429,753	*PAX3*	C/T substitution	C/C	C/C	C/C	C/C	C/C	[24, 25]
Lavender foal syndrome (LFS)	1	138,235,715	*MYO5A*	1 bp deletion: frameshift/stop	Not detected	Not detected	Not detected	Not detected	Not detected	[10]
Lethal white foal syndrome	17	50,624,771	*EDNRB*	AG deletion-insertion: Ile/Lys	Not detected	Not detected	Not detected	Not detected	Not detected	[26]
Silver coat color, congenital eye disease	6	73,665,304	*PMEL17*	C/T substitution: Arg/Cys	C/C	C/C	C/C	C/C	C/C	[27]
Macchiato, hearing loss	16	20,103,081	*MITF*	T/C substitution: Asn/Ser	T/T	T/T	T/T	T/T	T/T	[24]

**Table 4:** **Investigation of known variants affecting disease traits**

Phenotype	ECA	Position	Gene	Associated genotype	Genotype Duelmener	Genotype Arabian	Genotype Sorraia	Genotype Hanoverian 1	Genotype Hanoverian 2	References
Severe combined immunodeficiency	9	35,528,429	*DNAPK*	5bp deletion: stop	Not detected	Not detected	Not detected	Not detected	Not detected	[28]
Cerebellar abiotrophy	2	13,074,277	*TOE1*	G/A substitution: Arg/His	G/G	G/A	G/G	G/G	G/G	[15]
Glycogen branching enzyme deficiency	26	8,217,062	*GBE1*	C/A substitution: Tyr/stop	C/C	C/C	C/C	C/C	C/C	[29]
Equine hyperkalemic periodic paralysis	11	15,500,439	*SCN4A*	C/G substitution: Phe/Leu	C/C	C/C	C/C	C/C	C/C	[30]
Polysaccharide storage myopathy (PSSM type 1)	10	18,940,324	*GYS1*	G/A substitution: Arg/His	G/G	G/G	G/G	G/G	G/G	[31]
Malignant hyperthermia	10	9,554,699	*RYR1*	C/G substitution: Arg/Gly	C/C	C/C	C/C	C/C	C/C	[32]
Hereditary equine regional dermal asthenia	1	128,056,748	*PPIB*	G/A substitution: Gly/Arg	G/G	G/G	G/G	G/G	G/G	[33, 34]
Junctional epidermolysis bullosa	8	45,603,643	*LAMC2*	C insertion: frameshift/stop	Not detected	Not detected	Not detected	Not detected	Not detected	[35, 36]
Foal immunodeficiency syndrome	26	30,660,224	*SLC5A3*	C/T substitution: Pro/Leu	C/C	C/C	C/C	C/C	C/C	[37]

**Table 5:** **Investigation of known variants affecting performance traits**

Phenotype	ECA	Position	Gene	Associated genotype	Genotype Duelmener	Genotype Arabian	Genotype Sorraia	Genotype Hanoverian 1	Genotype Hanoverian 2	References
Body size	3	105,547,002	*LCORL*	C/T substitution	T/T	T/T	T/T	C/T	C/T	[16–18]
Gait coordination	23	22,999,655	*DMRT3*	C/A substitution: Ser/stop	C/C	C/C	C/C	C/C	C/C	[38]
Racing distance	18	66,493,737	*MSTN*	C/T substitution	C/T	T/T	C/C	T/T	T/T	[19]
Racing performance	4	38969307	*PDK4*	C/A substitution	C/C	A/A	C/C	A/C	C/C	[39]
Racing performance	4	38973231	*PDK4*	G/A substitution	A/A	A/A	G/G	A/G	A/G	[39]
Racing performance	10	15884567	*CKM*	G/A substitution	A/G	G/G	G/G	A/G	G/G	[40]
Racing performance	22	22684390	*COX4I2*	C/T substitution	C/C	C/T	T/T	C/T	C/C	[40]

## Discussion and conclusion

The objective of this study was to give an insight into the diversity of the horse population by comparative analysis of breed to non-breed horses using next generation sequencing. Both groups revealed a large number of potential novel SNPs and indels that could not be found in any horse database. Comparative analyses with BeadChip data and known variants causing coat color and disease phenotypes suggested that our sequencing data with a mean coverage of 11-25X provide a reliable basis for variant detection despite possible limitations due to lower sequence coverage in specific regions and errors in the reference genome that might simulate genetic variants [11]. Variant effect prediction by SNPEff was probably under the same limitations regarding the genome build EquCab2.70, but the use of this tool on basis of pre-build databases has been successfully applied for re-sequencing studies in horses and other mammals so far [41–43].

Our next generation sequencing data of one or two horses of different populations each gives us a broad idea of how diverse domestic horses really are. The analyzed horses are derived from different developmental groups of the horse population representing one of the oldest and strongly selected breeds (Arabian), one highly selected sport horse breed (Hanoverian) and two populations from different geographic backgrounds that underlie a strong natural selection (Duelmener, Sorraia). These horses were chosen as characteristic representatives for their population in order to reflect distinctive attributes of selectively bred horses in comparison to horse populations not bred for specific purposes. The use of the term “breed” has been discussed very differently. The main point for the differentiation of non-breed and breed was the question if the population was subject to controlled breeding and husbandry imposed by humans [44]. We suggest that the Duelmener and Sorraia populations are not exposed to those strong human influences and should therefore be grouped as non-breed.

In comparison to breed horses we were able to detect a larger number of private SNPs in non-breed horses. Nevertheless, despite the lower number of private SNPs, breed horses showed a large number of private indels with codon changing effects in genes involved in immune system processes. Some of these genes play a role in the cell surface receptor linked signal transduction (*olfactory receptor gene 56A4, OR56A4)*, others affect antigen processing and presentation (*SRSF protein kinase 2, SRPK2; CALPAIN-8, CAPN8*) [45–47]. The heterozygosity of variants in these immunity-related regions could be shown to be high in comparison to the detected variants of non-breed horses. Comparative analysis of modern domestic horses with a Przewalski horse suggested that regions of significant reduction of the genetic diversity in modern horses correspond to specifically selected loci while regions of high densities of mutations give evidence of continuous selection as it could be shown in immunity-related and olfactory genes in modern horses [8]. Our data confirm this assumption which presumably reflects the importance for variability of the immune system especially in breed horses [48]. In contrast to that, the investigated non-breed horses showed an enrichment of private mutations in genes affecting metabolism, anatomical structures, morphogenesis and cellular components which might give us an idea of the genetic background of their characteristics. We can only speculate if private mutations detected in pheromone and odorant binding *vomeronasal receptor 1 (EQUCABV1R928)* or in the *probable cation-transporting ATPase 13A4* (*ATP13A4)* as well as *ATPase, class VI, type 11B* (*ATP11B),* that play a role for chemoreception, might be involved in non-breed specific abilities. Nevertheless, despite similar characteristics to the Duelmener, the heterozygosity was inferior in the Sorraia horse in comparison with all other horses. We assume that this result is consistent with previous assumption that the Sorraia population shows losses in the level of heterozygosity as it has undergone a genetic bottleneck [49].

In conclusion, our analysis is provided to give an insight into possible interrelations between populations and specific characteristics. We suggest that this data of five horses form a basis for future verification studies in a larger number of horses that will build on these results and help to elucidate specific genetic features by further elimination of individual variants and by increased sequence coverage. Although we cannot exclude that some private mutations could be due to false detection or might be individual mutations for the analyzed horses we suppose that our data generally reflect the specific characteristics of breeds and non-breeds and give an idea of what the main developmental focus of each group is.

## Methods

### Ethics statement

All animal work has been conducted according to the national and international guidelines for animal welfare. The Lower Saxony state veterinary office at the Niedersächsisches Landesamt für Verbraucherschutz und Lebensmittelsicherheit, Oldenburg, Germany, was the responsible Institutional Animal Care and Use Committee (IACUC) for this specific study. The EDTA-blood sampling for the present study had been approved by the IACUC of Lower Saxony, the state veterinary office Niedersächsisches Landesamt für Verbraucherschutz und Lebensmittelsicherheit, Oldenburg, Germany (registration number 11A 160/7221.3-2.1-015/11, 8.84-02.05.20.12.066).

### Animals

Genomic DNA of a Duelmener mare, a Sorraja stallion, an Arabian stallion and two Hanoverian stallions was isolated using 600 ul EDTA blood which was drawn from one jugular vein with a sterile Vacuette system (Greiner Bio-One, Kremsmünster, Austria). An ethanol fraction was performed by 6 M NaCl, 70% ethanol, and 100% ethanol (Carl Roth, Karlsruhe, Germany) in consecutive steps according to standard protocols.

### Sequencing and alignment

Sequencing of the whole genome was performed using an Illumina HiSeq2000 (Illumina, San Diego, CA). The short-insert paired-end libraries were prepared using Illumina DNA sample preparation kit (Illumina) following manufacturer’s guidelines with minor modifications. Genomic DNA was quantified using Qubit 2.0 Fluorometer (Life Technologies, Eugene, Oregon) and 2.0 micrograms of genomic DNA were sheared on a Covaris E220 (Covaris, Woburn, MA), size selected and concentrated using AMPure XP beads (Agencourt, Beckman Coulter) in order to reach the fragment size of 220-480 bp. The fragmented DNA was end-repaired, adenylated and ligated to Illumina specific paired-end adaptors. The quantification of all libraries was done using the Library Quantification Kit (Kapa Biosystems, Woburn, MA). Four libraries were sequenced on one lane each and one library (Hanoverian 1) on two lanes of HiSeq2000 flowcell v3 (Illumina) in paired end mode (2 × 101 bp reads) using TruSeq SBS Kit v3-HS reagents (Illumina). Sequencing was performed according to standard Illumina operation procedures with minimal yield of 25 Gb for each sample.

Primary data analysis was carried out with the standard Illumina pipeline. Sequencing reads were trimmed from the end of the read until the first base over Q10 and reads shorter than 40 bp were discarded. The Genome Multitool (GEM) [50] mapper was used to identify all alignments to the reference (EquCab 2.70) with 4 or less mismatches which included up to one insertion or deletion of up to 25 bases. To map reads with higher divergence we additionally performed alignment with BFAST [51].

### Variant detection

For the discovery of SNPs and indels, we applied the Genome Analysis Toolkit (GATK) version 2.7-2 [52, 53]. Further basic statistics like the total read depth for each position, consensus quality, allele frequency and genotype quality for the variant in one sample were computed by SAMtools (Sequence Alignment/Map) and BCFtools from the SAMtools package [54]. The chromosomal enrichment of detected variants was accounted dividing the number of variants by the length of the chromosome (bp) in order to compute the average number of variants per base pair. The distribution of variants of the whole-genome was computed dividing the total number of variants by the total length of reference genome and used as threshold value for significant chromosomal enrichment. All experiment files are available at the NCBI Sequence Read Archive (http://www.ncbi.nlm.nih.gov/sra), accession number SRP033361. The VCF file can be downloaded at Intrepid Bioinformatics at http://dx.doi.org/10.13013/J6MW2F2B.

### Comparative analyses

For comparative analysis with BeadChip data of the Arabian and Hanoverians we genotyped 50 ng/μl DNA on the Illumina Equine SNP50 genotyping BeadChip (Illumina) for 54,602 SNPs using standard procedures as recommended by the manufacturer. Data were analyzed and file clusters were generated with the genotyping module version 3.2.32 of the BeadStudio program [17] (Illumina). All genotyped SNPs were compared to our next generation sequencing data using SAS/Genetics, version 9.4 (Statistical Analysis System, Cary, NC, 2013) and complementary bases were adjusted for comparison. In a second step we performed SAS-analysis to identify known and novel variants in comparison with the databases dbSNP (ftp://ftp.ncbi.nih.gov/snp/organisms/horse_9796/chr_rpts/, download 03.09.2009), Broad Institute (http://www.broadinstitute.org/ftp/distribution/horse_snp_release/v2/, download 19.09.2013), Ensembl (ftp://ftp.ensembl.org/pub/release-73/variation/gvf/equus_caballus/ Equus_caballus.gvf.gz, download 19.09.2013) and published data from Orlando *et al.* [8] and Doan *et al.* [11] *.* A concordance of our detected variants with these data was calculated by the position of the variants in the genome.

### Functional annotation and analysis

We performed functional annotation of the detected variants using the genetic variant annotation and effect prediction toolbox SNPEff [13], version 3.1 that provided lists of expected effects, their position and involved genes affected by these variants. The variants were categorized by their functional class and impact. Finally, the data of calculated effects produced in known genes were provided for further analysis in a VCF output file. We further categorized this data by their influence on coding or regulative regions in breed and non-breed horses. All genes were converted to human ortholog genes using g: Profiler [55, 56] in order to improve the identification of gene ontology terms. The raw and Bonferroni corrected P-values for the enrichment analysis of genes involved in biological processes were computed using PANTHER (Protein ANalysis THrough Evolutionary Relationships, version 8.0) classification system [57, 58]. Values for heterozygosities were defined for all 10,193,421 sites with SNPs. In addition, we calculated heterozygosities for regions with private variants in immunity related genes using SAS/Genetics.

## Electronic supplementary material

Additional file 1: **Summary of next generation sequencing data of five horses on the Illumina HiSeq2000.** The mapping metrics **(** **A** **)** mean and median coverage **(** **B** **)** and number of shared and individual variants **(** **C** **)** are shown. (DOCX 18 KB)

Additional file 2: **Total number of variants by chromosome detected by next generation sequencing in breed and non-breed horses.** (TIFF 427 KB)

Additional file 3: **Average number of variants per base pair by chromosome (ECA) detected by next generation sequencing of five horses.** ECA12 and ECA19 show the highest number of detected variants with regard to the chromosomal size. (TIFF 409 KB)

Additional file 4: **Comparison of non exonic SNPs and indels from current analysis with known variants from different databases.** The total number of SNPs and indels per SNPEff term detected in five horses and their concordance with dbSNP, Broad Institute and Ensembl data as well as data published by Orlando et al. [8] and Doan et al. [11] are shown. (DOCX 17 KB)

Additional file 5: **Functional classification analysis of the predicted private non-synonymous SNPs in non-breed** **(** **A** **)** **and breed** **(** **B** **)** **horses.** Both groups show a similar distribution of gene functions. (JPEG 433 KB)

Additional file 6: **Number of effects by private and shared indels detected by next generation sequencing in five horses.** The presented results are classified by SNPEff terms for each breed. (DOCX 16 KB)

Additional file 7: **Characterization of private variations with possibly damaging effects.** Private SNPs and INDELs which are predicted to cause a loss of stop codon, exon deletions as well as codon changes are shown. (DOCX 49 KB)

Additional file 8: **Functional classification analysis of the predicted codon changes possibly caused by private indels.** Genes involved in immune system processes are more frequent in breed horses (22.6%) in comparison with non-breed horses (6.7%). (JPEG 420 KB)

Additional file 9: **Enrichment analysis of significantly overrepresented genes involved in biological processes.** The software PANTHER was used for the evaluation of SNPs in coding regions and regulative regions for non-breed and breed horses. Raw P-values and Bonferoni corrected significant P-values are shown. (DOCX 32 KB)

## Abbreviations

ASIP: Agouti-signaling protein precursor
ATP13A4: Probable cation-transporting ATPase 13A4
CKM: Creatine kinase M-type
cm: Centimeter
CNDP2: Dipeptidase 2, metallopeptidase M20 family
COX4I2: Cytochrome c oxidase subunit 4 isoform 2
DMRT3: Doublesex and mab-3 related transcription factor 3
DNAPK: DNA-dependent protein kinase catalysic subunit
EDNRB: Endothelin B receptor precursor
GBE1: Glucan (1, 4-alpha-), branching enzyme 1
GYS1: Glycogen synthase muscle
indel: Insertion/deletion
LAMC2: Laminin subunit gamma-2 precursor
LCORL: Ligand dependent nuclear receptor corepressor-like
MC1R: Melanocyte-stimulating hormone receptor
MITF: Microphthalmia-associated transcription factor
MSTN: Myostatin gene
MYO5A: Myosin-Va isoform 1
NGS: Next generation sequencing
OR2A2: Olfactory receptor, family 2, subfamily A, member 2
PAX3: Paired box 3
PDK4: Pyruvate dehydrogenase kinase isozyme 4
PMEL17: Melanocyte protein 17 precursor
PPIB: Peptidyl-prolyl cis-trans isomerase B
RTDR1: Rhabdoid tumor deletion region gene 1
RYR1: Ryanodine receptor 1 isoform 2
SCN4A: Sodium channel protein type 4 subunit alpha
SLC36A1: Proton-coupled amino acid transporter 1
SLC45A2: Membrane-associated transporter protein isoform
SLC5A3: Solute carrier family 5 member 3
SNP: Single nucleotide polymorphism
STX17: Syntaxin-17
TOE1: Target of EGR1
TRBV6-4: T cell receptor beta variable 6–4.
