# Supplementary material for: Next generation sequencing gives an insight into the characteristics of highly selected breeds versus non-breed horses in the course of domestication
Source: BMC Genomics. 2014 Jul 4;15(1):562. doi: 10.1186/1471-2164-15-562 (PMC4097168; doi:10.1186/1471-2164-15-562)
Supplement: Supplementary file 1 — Additional file 1: Summary of next generation sequencing data of five horses on the Illumina HiSeq2000. The mapping metrics ( A ) mean and median coverage ( B ) and number of shared and individual variants ( C ) are shown. (DOCX 18 KB) [file 12864_2013_6235_MOESM1_ESM.docx]

Additional file 1. Summary of next generation sequencing data of five horses on the Illumina HiSeq2000. The mapping metrics (A), mean and median coverage (B) and number of shared and individual variants (C) are shown.

Additional file 1A.

|  | Duelmener | Arabian | Sorraia | Hanoverian 1 | Hanoverian 2 |
| --- | --- | --- | --- | --- | --- |
| Number of lanes | 1 | 1 | 1 | 2 | 1 |
| **Yield summary** |  |  |  |  |  |
| Passing filter reads | 200,443,209 | 192,811,044 | 178,298,051 | 358,675,899 | 158,295.662 |
| Read lengths | 100/100 | 100/100 | 100/100 | 100/100 | 100/100 |
| Yield total (bp) | 40,088,641,800 | 38,562,208,800 | 35,659,610,200 | 71,735,179,800 | 31,659,132,400 |
| Bases trimmed total (bp) | 1,246,525,128 | 1,016,554,704 | 802,897,927 | 1,588,568,683 | 631,928,204 |
| Trimmed yield total (bp) | 38,823,728,068 | 37,539,992,329 | 34,851,643,176 | 70,136,418,921 | 31,022,673,947 |
| **Mapping summary** |  |  |  |  |  |
| Uniquely mapping reads | 313,601,649 | 302,312,458 | 275,750,885 | 578,405,001 | 248,223,628 |
| Multiply mapping reads | 35,836,370 | 36,069,183 | 33,469,110 | 59,600,910 | 28,183,136 |
| Unmapped reads | 26,806,845 | 27,591,632 | 29,823,757 | 46,709,523 | 25,284,257 |
| Ambiguous reads | 24,641,554 | 19,648,815 | 17,552,350 | 32,636,364 | 14,900,303 |
| Split mapped reads | 1,331,824 | 1,237,694 | 1,354,839 | 2,205,267 | 879,636 |

Additional file 1B.

|  | Duelmener | Arabian | Sorraia | Hanoverian 1 | Hanoverian 2 |
| --- | --- | --- | --- | --- | --- |
| Percentage of based with at least 10x coverage | 67.38 | 62.54 | 52.87 | 96.65 | 40.86 |
| Mean coverage | 14.02 | 13.37 | 12.21 | 25.38 | 10.97 |
| Median coverage | 13.00 | 12.00 | 11.00 | 24.00 | 10.00 |
|  |  |  |  |  |  |

Additional file 1C.

| Variant | all | shared | Duelmener | Arabian | Sorraia | Hannoverian 1 | Hannoverian 2 |
| --- | --- | --- | --- | --- | --- | --- | --- |
| SNP | 10193421 | 1712330 | 5391494 | 5156659 | 5156659 | 5264058 | 5032162 |
| INDEL | 1361948 | 551444 | 935333 | 905581 | 905581 | 931276 | 889106 |
| Total variants | 11555369 | 2263774 | 6326827 | 6062240 | 6062240 | 6195334 | 5921268 |
