# Supplementary figures and images for: Next generation sequencing gives an insight into the characteristics of highly selected breeds versus non-breed horses in the course of domestication
Source: BMC Genomics. 2014 Jul 4;15(1):562. doi: 10.1186/1471-2164-15-562 (PMC4097168; doi:10.1186/1471-2164-15-562)

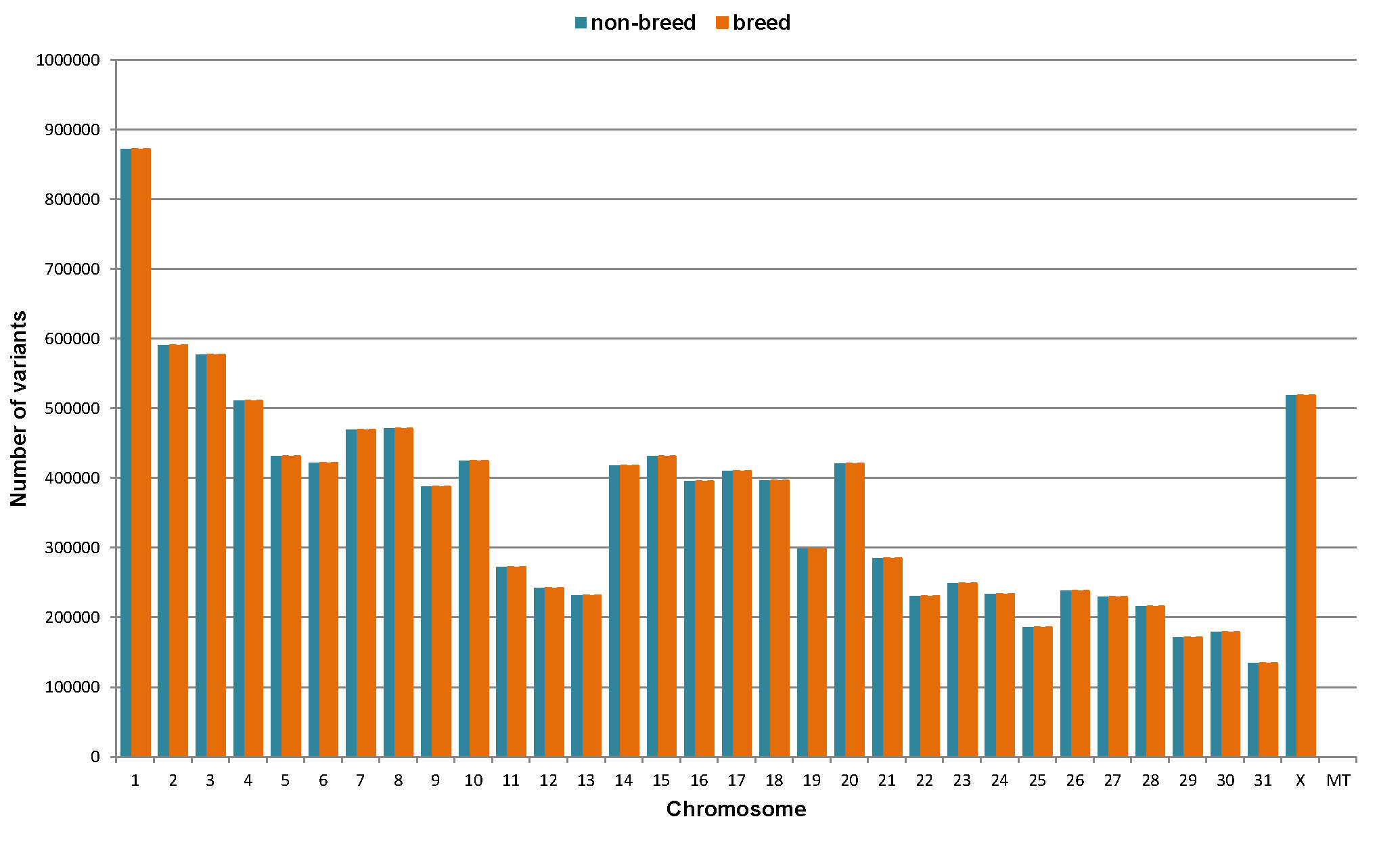

Supplement: Supplementary file 2 — Additional file 2: Total number of variants by chromosome detected by next generation sequencing in breed and non-breed horses. (TIFF 427 KB) [file 12864_2013_6235_MOESM2_ESM.tiff]

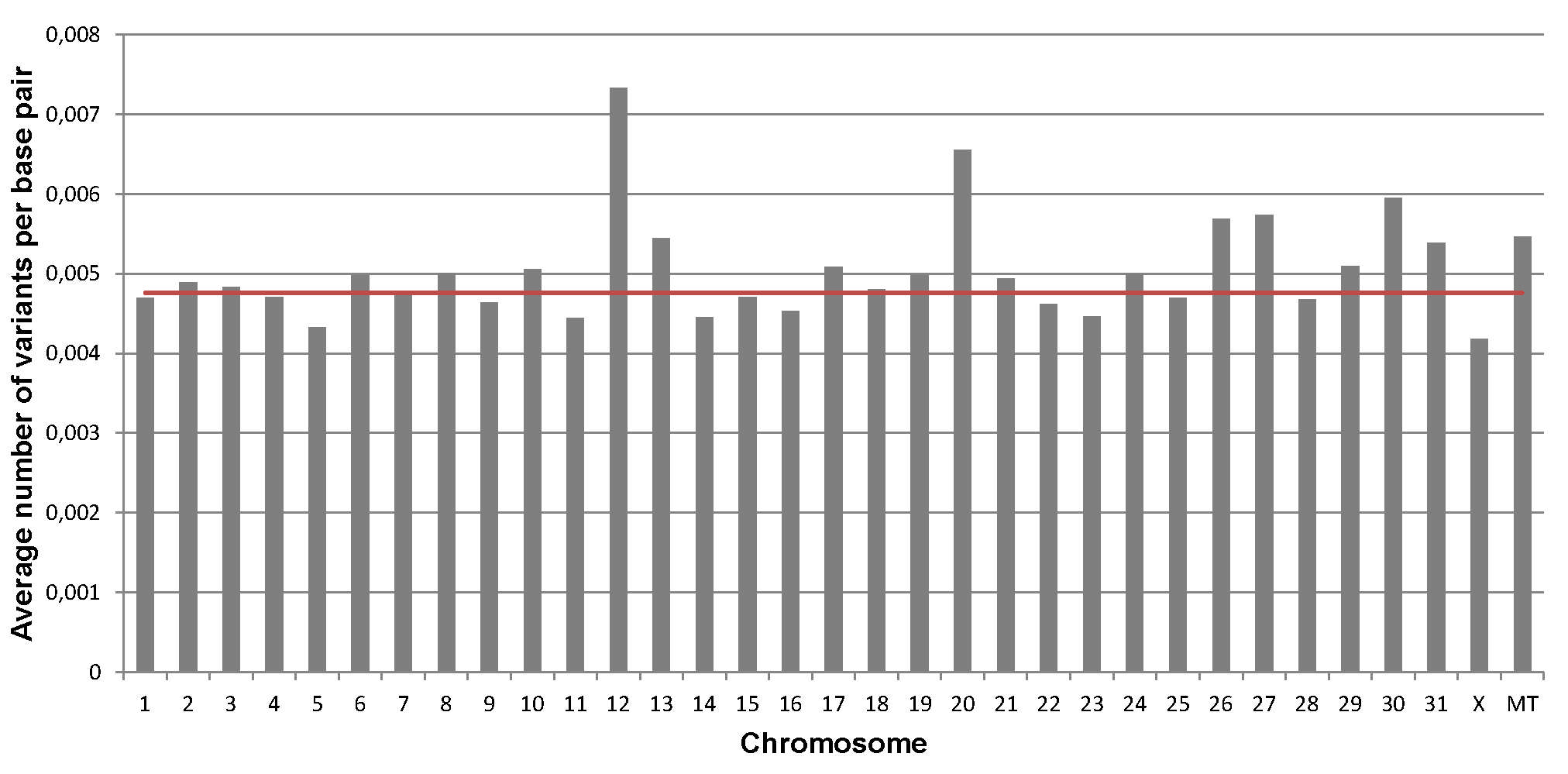

Supplement: Supplementary file 3 — Additional file 3: Average number of variants per base pair by chromosome (ECA) detected by next generation sequencing of five horses. ECA12 and ECA19 show the highest number of detected variants with regard to the chromosomal size. (TIFF 409 KB) [file 12864_2013_6235_MOESM3_ESM.tiff]

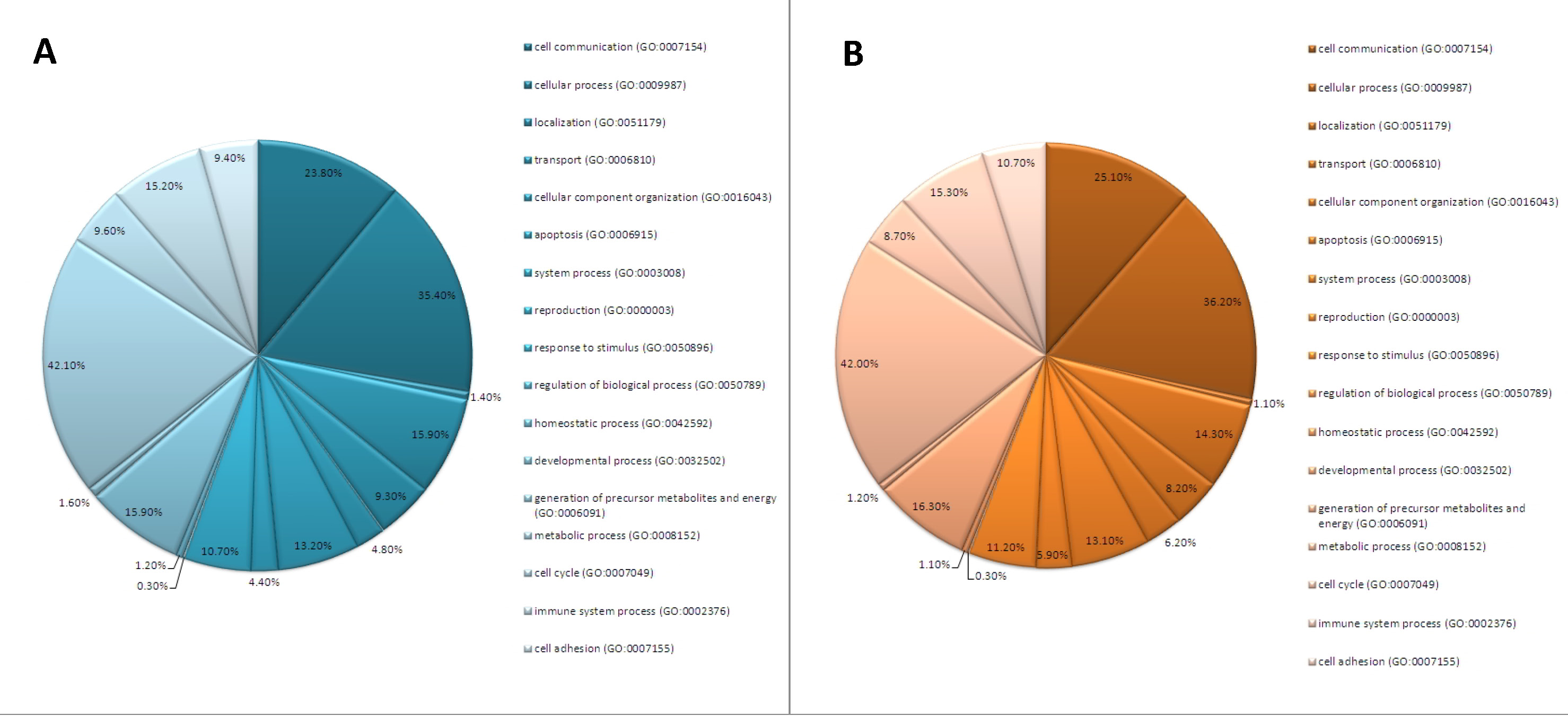

Supplement: Supplementary file 5 — Additional file 5: Functional classification analysis of the predicted private non-synonymous SNPs in non-breed ( A ) and breed ( B ) horses. Both groups show a similar distribution of gene functions. (JPEG 433 KB) [file 12864_2013_6235_MOESM5_ESM.jpeg]

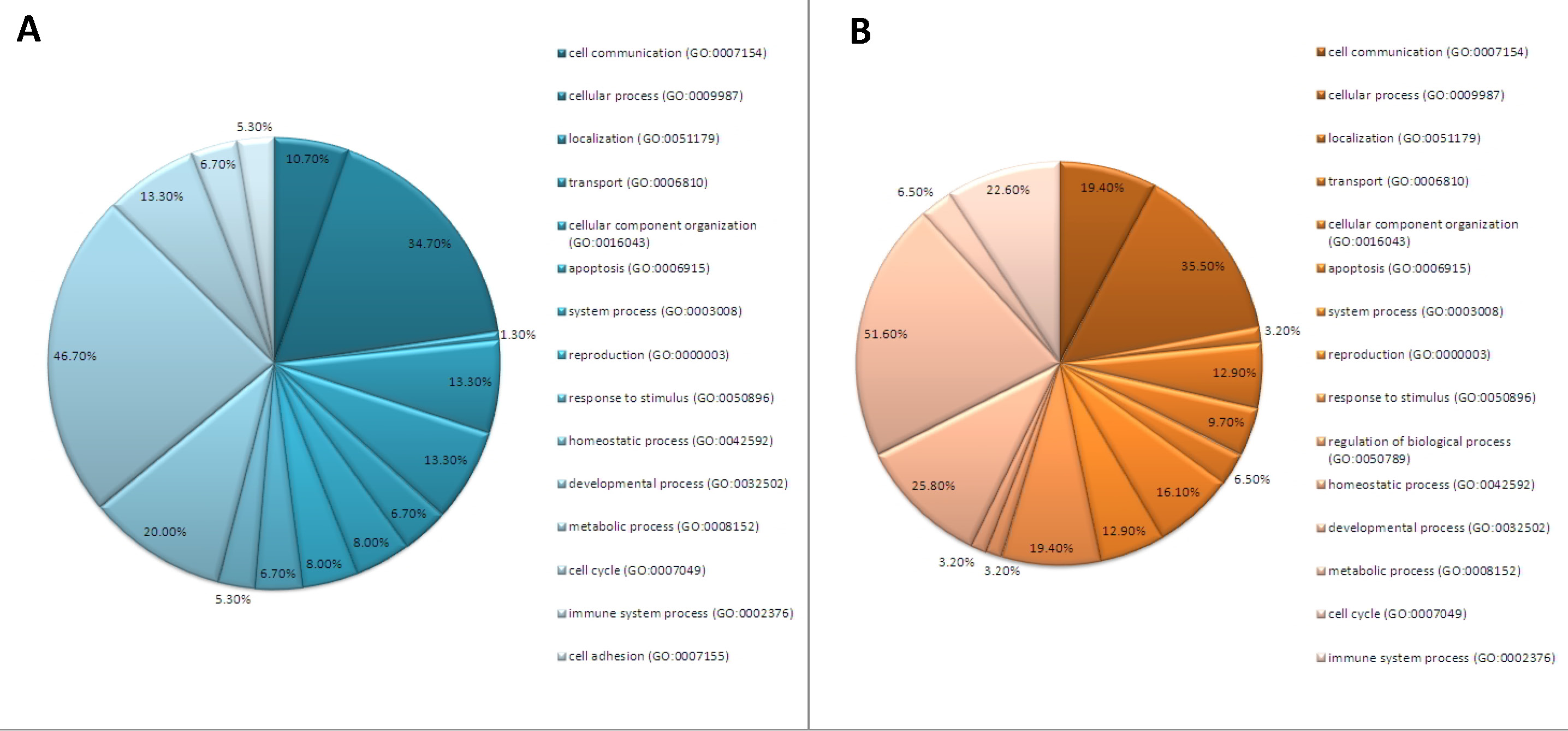

Supplement: Supplementary file 8 — Additional file 8: Functional classification analysis of the predicted codon changes possibly caused by private indels. Genes involved in immune system processes are more frequent in breed horses (22.6%) in comparison with non-breed horses (6.7%). (JPEG 420 KB) [file 12864_2013_6235_MOESM8_ESM.jpeg]
