# Supplementary material for: Next generation sequencing gives an insight into the characteristics of highly selected breeds versus non-breed horses in the course of domestication
Source: BMC Genomics. 2014 Jul 4;15(1):562. doi: 10.1186/1471-2164-15-562 (PMC4097168; doi:10.1186/1471-2164-15-562)
Supplement: Supplementary file 4 — Additional file 4: Comparison of non exonic SNPs and indels from current analysis with known variants from different databases. The total number of SNPs and indels per SNPEff term detected in five horses and their concordance with dbSNP, Broad Institute and Ensembl data as well as data published by Orlando et al. [8] and Doan et al. [11] are shown. (DOCX 17 KB) [file 12864_2013_6235_MOESM4_ESM.docx]

Additional file 4. Comparison of non exonic SNPs and indels from current analysis with known variants from different databases. The total number of SNPs and indels per SNPEff term detected in five horses and their concordance with dbSNP, Broad Institute and Ensembl data as well as data published by Orlando *et al.* [8] and Doan *et al.* [11] are shown.

| SNPEff terms by type | Total | Common SNPs with dbSNP | Common SNPs with ensembl | Common SNPs with Broad | Common variants with Orlando *et al.* [8] | Common variants with Doan *et al.* [11] | Novel variants |
| --- | --- | --- | --- | --- | --- | --- | --- |
| SNPs |  |  |  |  |  |  |  |
| Downstream | 560312 | 48917 | 48857 | 49125 | 352302 | 135493 | 184569 |
| Intergenic | 7452057 | 657456 | 657709 | 660233 | 4712601 | 1439956 | 2511705 |
| Intron | 3392186 | 311490 | 311342 | 311859 | 2195097 | 757999 | 1085722 |
| Splice site acceptor | 359 | 19 | 19 | 19 | 176 | 98 | 151 |
| Splice site donor | 552 | 23 | 21 | 23 | 243 | 141 | 267 |
| Start gained | 619 | 36 | 36 | 36 | 282 | 167 | 288 |
| Upstream | 598514 | 49944 | 49764 | 50074 | 362119 | 147132 | 205643 |
| 3‘UTR | 7012 | 607 | 603 | 599 | 4061 | 1543 | 2667 |
| 5‘UTR | 4135 | 154 | 154 | 154 | 1755 | 1450 | 1782 |
| indels |  |  |  |  |  |  |  |
| Downstream | 87856 | - | - | - | 29457 | 1930 | 56491 |
| Intergenic | 971326 | - | - | - | 336700 | 12717 | 622077 |
| Intron | 486004 | - | - | - | 172930 | 7352 | 305804 |
| Splice site acceptor | 1645 | - | - | - | 600 | 100 | 945 |
| Splice site donor | 1781 | - | - | - | 616 | 138 | 1027 |
| Upstream | 99739 | - | - | - | 30570 | 2854 | 66339 |
| 3‘UTR | 1324 | - | - | - | 443 | 48 | 833 |
| 5‘UTR | 1984 | - | - | - | 396 | 125 | 1463 |
