# Supplementary material for: Next generation sequencing gives an insight into the characteristics of highly selected breeds versus non-breed horses in the course of domestication
Source: BMC Genomics. 2014 Jul 4;15(1):562. doi: 10.1186/1471-2164-15-562 (PMC4097168; doi:10.1186/1471-2164-15-562)
Supplement: Supplementary file 6 — Additional file 6: Number of effects by private and shared indels detected by next generation sequencing in five horses. The presented results are classified by SNPEff terms for each breed. (DOCX 16 KB) [file 12864_2013_6235_MOESM6_ESM.docx]

Additional file 6. Number of effects by private and shared indels detected by next generation sequencing in five horses. The presented results are classified by SNPEff terms for each breed.

| SNPEff terms by type | Shared  (all 5 horses) | Duelmener | Sorraia | Arabian | Hanoverian  (two horses) |
| --- | --- | --- | --- | --- | --- |
| Codon change plus codon deletion | 19 | 10 | 10 | 10 | 1 |
| Codon change plus codon insertion | 80 | 10 | 5 | 6 | 4 |
| Codon deletion | 31 | 24 | 6 | 16 | 5 |
| Codon insertion | 67 | 7 | 3 | 1 | 0 |
| Downstream | 33108 | 3723 | 3097 | 2646 | 1376 |
| Exon | 466 | 44 | 30 | 22 | 12 |
| Exon_Deleted | 0 | 1 | 0 | 0 | 0 |
| Frameshift | 5708 | 44 | 33 | 39 | 13 |
| Intergenic | 383948 | 53282 | 43356 | 37643 | 971326 |
| Intron | 159948 | 20434 | 16803 | 14676 | 7881 |
| Splice site acceptor | 1330 | 5 | 5 | 4 | 4 |
| Splice site donor | 1450 | 5 | 5 | 4 | 4 |
| Start lost | 4 | 0 | 0 | 0 | 0 |
| Stop gained | 10 | 0 | 0 | 0 | 0 |
| Upstream | 45219 | 3648 | 3079 | 2648 | 1373 |
| 3‘UTR | 639 | 47 | 57 | 31 | 17 |
| 5‘UTR | 1610 | 20 | 12 | 18 | 9 |
| total | 629686 | 81304 | 66501 | 57764 | 982025 |
