# Supplementary material for: Next generation sequencing gives an insight into the characteristics of highly selected breeds versus non-breed horses in the course of domestication
Source: BMC Genomics. 2014 Jul 4;15(1):562. doi: 10.1186/1471-2164-15-562 (PMC4097168; doi:10.1186/1471-2164-15-562)
Supplement: Supplementary file 7 — Additional file 7: Characterization of private variations with possibly damaging effects. Private SNPs and INDELs which are predicted to cause a loss of stop codon, exon deletions as well as codon changes are shown. (DOCX 49 KB) [file 12864_2013_6235_MOESM7_ESM.docx]

Additional file 7. Characterization of private variations with possibly damaging effects. Private SNPs and INDELs which are predicted to cause a loss of stop codon, exon deletions as well as codon changes are shown.

| ECA | Position (bp) | Genotype  Duelmener | Arabian | Sorraia | Hanoverian 1 | Hanoverian 2 | Gene  (human orthologue) | Function |
| --- | --- | --- | --- | --- | --- | --- | --- | --- |
| Loss of stop codon | |  |  |  |  |  |  |  |
| 19 | 30,336,306 | C/T | T/T | T/T | T/T | T/T | *ATP13A4* | *Probable cation-transporting ATPase 13A4*; calcium regulation |
| 4 | 95,114,829 | G/G | G/G | C/G | G/G | G/G | *ENSECAG00000000628 (TRBV6-4)* | Novel gene;  *T cell receptor beta variable 6-4* |
| 4 | 97,312,069 | T/T | T/T | C/T | T/T | T/T | *ENSECAG00000007186 (OR2A2)* | Novel gene;  *olfactory receptor, family 2, subfamily A, member 2* |
| 8 | 697,117 | T/T | T/T | T/T | G/T | G/T | *RTDR1* | *Rhabdoid tumor deletion region gene 1*;  Often deleted in pediatric rhabdoid tumors |
| 10 | 11,712,077 | T/T | T/T | T/T | C/T | C/T | *ENSECAG00000008382* | Novel gene |
| Exon deletion | |  |  |  |  |  |  |  |
| 11 | g. 23893287 del | del/ref | ref/ref | ref/ref | ref/ref | ref/ref | ENSECAG00000018401 (*CNDP2*) | *CNDP dipeptidase 2 (metallopeptidase M20 family)* |

Additional file 7 continued.

| ECA | Position (bp) | Genotype  Duelmener | Arabian | Sorraia | Hanoverian 1 | Hanoverian 2 | Gene  (human/ mouse orthologue) | Function |
| --- | --- | --- | --- | --- | --- | --- | --- | --- |
| Codon change plus codon deletion | | | |  |  |  |  |  |
| 3 | g.10144936del | del/ref | ref/ref | ref/ref | ref/ref | ref/ref | *MMP15* | Matrix metallopeptidase 15 (membrane-inserted) |
| 7 | g.74301328del | del/ref | ref/ref | ref/ref | ref/ref | ref/ref | *ENSECAG00000013241 (4931431F19Rik)* | Novel gene  RIKEN cDNA 4931431F19 gene |
| 8 | g.54314078del | del/ref | ref/ref | ref/ref | ref/ref | ref/ref | *ENSECAG00000015851*  *(ASXL3)* | Novel gene  additional sex combs like 3 (Drosophila) |
| 10 | g.21914712del | del/ref | ref/ref | ref/ref | ref/ref | ref/ref | *EQUCABV1R928* | Vomeronasal 1 receptor equCabV1R928 |
| 11 | g.21176221del | del/ref | ref/ref | ref/ref | ref/ref | ref/ref | *KRT16* | Keratin 16 |
| 25 | g.26462600del | del/del | ref/ref | ref/ref | ref/ref | ref/ref | *ENSECAG00000002846 (OR1N2)* | Novel gene  olfactory receptor, family 1, subfamily N, member 2 |
| 25 | g.30408177del | del/ref | ref/ref | ref/ref | ref/ref | ref/ref | *ZBTB43* | Zinc finger and BTB domain containing 43 |
| 26 | g.41057486del | del/ref | ref/ref | ref/ref | ref/ref | ref/ref | *COL6A1* | Collagen, type VI, alpha 1 |
| 29 | g.21584771del | del/ref | ref/ref | ref/ref | ref/ref | ref/ref | *ENSECAG00000024294* | Novel gene |
| 32 | g.11570775del | del/ref | ref/ref | ref/ref | ref/ref | ref/ref | *MAGEB17* | Melanoma antigen family B, 17 |

Additional file 7 continued.

| ECA | Position (bp) | Genotype  Duelmener | Arabian | Sorraia | Hanoverian 1 | Hanoverian 2 | Gene  (human/ mouse orthologue) | Function |
| --- | --- | --- | --- | --- | --- | --- | --- | --- |
| Codon change plus codon insertion | | | | | |  |  |  |
| 6 | g.34173309ins | del/ref | ref/ref | ref/ref | ref/ref | ref/ref | *ZNF384* | Zinc finger protein 384 |
| 7 | g.73725671ins | del/ref | ref/ref | ref/ref | ref/ref | ref/ref | *ENSECAG00000022991 (Olfr632)* | Novel gene, olfactory receptor 632 |
| 7 | g.75464491ins | del/ref | ref/ref | ref/ref | ref/ref | ref/ref | *ENSECAG00000000811 (TIMM10B)* | Novel gene, translocase of inner mitochondrial membrane 10 homolog B (yeast) |
| 8 | g.21927552ins | del/ref | ref/ref | ref/ref | ref/ref | ref/ref | *RNF34* | Ring finger protein 34, E3 ubiquitin protein ligase |
| 10 | g.18867613ins | del/del | ref/ref | ref/ref | ref/ref | ref/ref | *PPP1R15A* | Protein phosphatase 1, regulatory subunit15A |
| 14 | g.27410907ins | del/ref | ref/ref | ref/ref | ref/ref | ref/ref | *SYNPO* | Synaptopodin |
| 18 | g.75327700ins | del/del | ref/ref | ref/ref | ref/ref | ref/ref | *SGOL2* | Shugoshin-like 2 (S. pombe) |
| 19 | g.33169014ins | del/ref | ref/ref | ref/ref | ref/ref | ref/ref | *C3orf43* | Single-pass membrane protein with coiled-coil domains 1 |
| 28 | g.35931195ins | del/ref | ref/ref | ref/ref | ref/ref | ref/ref | *APOBEC3Z1B* | apolipoprotein B mRNA editing enzyme, catalytic polypeptide-like 3B |
| 32 | g.39615972ins | del/ref | ref/ref | ref/ref | ref/ref | ref/ref | *PRICKLE3* | Prickle homolog 3 (Drosophila) |

Additional file 7 continued.

| ECA | Position (bp) | Genotype  Duelmener | Arabian | Sorraia | Hanoverian 1 | Hanoverian 2 | Gene  (human/ mouse orthologue) | Function |
| --- | --- | --- | --- | --- | --- | --- | --- | --- |
| Codon deletion | | | |  |  |  |  |  |
| 1 | g.61408174del | del/ref | ref/ref | ref/ref | ref/ref | ref/ref | *SYNPO2L* | Synaptopodin 2-like |
| 1 | g.155196988del | del/ref | ref/ref | ref/ref | ref/ref | ref/ref | *ENSECAG00000004351* | Novel gene |
| 1 | g.162158020del | del/ref | ref/ref | ref/ref | ref/ref | ref/ref | *TGM1* | Transglutaminase 1 |
| 4 | g.94567160del | del/ref | ref/ref | ref/ref | ref/ref | ref/ref | *TAS2R3* | Taste receptor, type 2, member 3 |
| 4 | g.95201969del | del/ref | ref/ref | ref/ref | ref/ref | ref/ref | *ENSECAG00000005284 (*TRBV6-1) | T cell receptor beta variable 6-1 |
| 4 | g.105772440del | del/ref | ref/ref | ref/ref | ref/ref | ref/ref | *PAXIP1* | PAX interacting (with transcription-activation domain) protein 1 |
| 5 | g.23683163del | del/ref | ref/ref | ref/ref | ref/ref | ref/ref | *ENSECAG00000023432 (*L1TD1) | LINE-1 type transposase domain containing 1 |
| 5 | g.45266305del | del/ref | ref/ref | ref/ref | ref/ref | ref/ref | *TCHH (*FLG) | Novel gene, filaggrin |
| 6 | g.67333222del | del/ref | ref/ref | ref/ref | ref/ref | ref/ref | *PRPF40B* | PRP40 pre-mRNA processing factor 40 homolog B |
| 7 | g.72206683del | del/ref | ref/ref | ref/ref | ref/ref | ref/ref | *ENSECAG00000008050* | Novel gene |
| 8 | g.1516526del | del/ref | ref/ref | ref/ref | ref/ref | ref/ref | *ENSECAG00000012783* | Novel gene, Proteasome subunit alpha type |
| 8 | g.10907476del | del/ref | ref/ref | ref/ref | ref/ref | ref/ref | *SGSM1* | Small G protein signaling modulator 1 |
| 10 | g.9544928del | del/ref | ref/ref | ref/ref | ref/ref | ref/ref | *RYR1* | Ryanodine receptor 1 (skeletal) |

Additional file 7 continued.

| ECA | Position (bp) | Genotype  Duelmener | Arabian | Sorraia | Hanoverian 1 | Hanoverian 2 | Gene  (human/ mouse orthologue) | Function |
| --- | --- | --- | --- | --- | --- | --- | --- | --- |
| Codon deletion | |  |  |  |  |  |  |  |
| 10 | g.27934370del | del/ref | ref/ref | ref/ref | ref/ref | ref/ref | *A1BG* | Alpha-1-B glycoprotein |
| 10 | g.65279069del | del/ref | ref/ref | ref/ref | ref/ref | ref/ref | *ZUFSP* | Zinc finger with UFM1-specific peptidase domain |
| 11 | g.4215546del | del/ref | ref/ref | ref/ref | ref/ref | ref/ref | *SOCS3* | Suppressor of cytokine signaling 3 |
| 11 | g.21495399del | del/ref | ref/ref | ref/ref | ref/ref | ref/ref | *KRTAP4-1* | Keratin associated protein 4-1 |
| 12 | g.31758478del | del/ref | ref/ref | ref/ref | ref/ref | ref/ref | *PHRF1* | PHD and ring finger domains 1 |
| 13 | g.19299225del | del/ref | ref/ref | ref/ref | ref/ref | ref/ref | *TBC1D10B* | TBC1 domain family, member 10B |
| 16 | g.72829025del | del/ref | ref/ref | ref/ref | ref/ref | ref/ref | *DZIP1L* | DAZ interacting zinc finger protein 1-like |
| 20 | g.29691501del | del/del | ref/ref | ref/ref | ref/ref | ref/ref | *C6orf136* | Chromosome 6 open reading frame 136 |
| 25 | g.37576899del | del/ref | ref/ref | ref/ref | ref/ref | ref/ref | *SNAPC4* | Small nuclear RNA activating complex, polypeptide 4, 190kDa |
| 27 | g.21110508del | del/ref | ref/ref | ref/ref | ref/ref | ref/ref | *PCM1* | Pericentriolar material 1 |
| 28 | g.37745582del | del/ref | ref/ref | ref/ref | ref/ref | ref/ref | *L3MBTL2* | L(3)mbt-like 2 (Drosophila) |

Additional file 7 continued.

| ECA | Position (bp) | Genotype  Duelmener | Arabian | Sorraia | Hanoverian 1 | Hanoverian 2 | Gene  (human/ mouse orthologue) | Function |
| --- | --- | --- | --- | --- | --- | --- | --- | --- |
| Codon insertion | | |  |  |  |  |  |  |
| 6 | g.69356373ins | del/ref | ref/ref | ref/ref | ref/ref | ref/ref | *ENSECAG00000024398 (*KRT86) | keratin 86 |
| 11 | g.23394340ins | del/ref | ref/ref | ref/ref | ref/ref | ref/ref | *MLLT6* | Myeloid/lymphoid or mixed-lineage leukemia (trithorax homolog, Drosophila); translocated to, 6 |
| 13 | g.18419139ins | del/ref | ref/ref | ref/ref | ref/ref | ref/ref | *SLC5A2* | Solute carrier family 5 (sodium/glucose cotransporter), member 2 |
| 14 | g.2654877ibs | del/ref | ref/ref | ref/ref | ref/ref | ref/ref | *C5orf45* | Chromosome 5 open reading frame 45 |
| 19 | g.37152682ins | del/ref | ref/ref | ref/ref | ref/ref | ref/ref | *CASR* | Extracellular calcium-sensing receptor |
| 20 | g.56605821ins | del/ref | ref/ref | ref/ref | ref/ref | ref/ref | *PTP4A1* | Protein tyrosine phosphatase type IVA, member 1 |
| 24 | g.46211281ins | del/ref | ref/ref | ref/ref | ref/ref | ref/ref | *KIAA0284* | Centrosomal protein 170B |

Additional file 7 continued.

| ECA | Position (bp) | Genotype  Duelmener | Arabian | Sorraia | Hanoverian 1 | Hanoverian 2 | Gene  (human/ mouse orthologue) | Function |
| --- | --- | --- | --- | --- | --- | --- | --- | --- |
| Codon change plus codon deletion | | | | |  |  |  |  |
| 1 | g.157020495del | ref/ref | del/ref | ref/ref | ref/ref | ref/ref | *OR11H6* | Olfactory receptor, family 11, subfamily H, member 6 |
| 2 | g.53670326del | ref/ref | del/ref | ref/ref | ref/ref | ref/ref | *NEFM* | Neurofilament, medium polypeptide |
| 4 | g.83869332del | ref/ref | del/ref | ref/ref | ref/ref | ref/ref | *ENSECAG00000019209 (L1TD1)* | Novel gene  LINE-1 type transposase domain containing 1 |
| 10 | g.9605528del | ref/ref | del/ref | ref/ref | ref/ref | ref/ref | *RYR1* | Ryanodine receptor 1 (skeletal) |
| 12 | g.12318191del | ref/ref | del/ref | ref/ref | ref/ref | ref/ref | *PTPRJ* | Protein tyrosine phosphatase, receptor type, J |
| 13 | g.39217992del | ref/ref | del/ref | ref/ref | ref/ref | ref/ref | *SLX4* | SLX4 structure-specific endonuclease subunit |
| 15 | g.12339457del | ref/ref | del/ref | ref/ref | ref/ref | ref/ref | *ADRA2B* | Alpha-2B adrenergic receptor |
| 18 | g.55571065del | ref/ref | del/ref | ref/ref | ref/ref | ref/ref | *HNRNPA3* | Heterogeneous nuclear ribonucleoprotein A3 |
| 24 | g.46286579del | ref/ref | del/ref | ref/ref | ref/ref | ref/ref | *C14orf79* | Chromosome 14 open reading frame 79 |
| 30 | g.9438048del | ref/ref | del/ref | ref/ref | ref/ref | ref/ref | *CAPN8* | Calpain 8 |
| Codon change plus codon insertion | | | |  |  |  |  |  |
| 2 | g.25180860ins | ref/ref | del/ref | ref/ref | ref/ref | ref/ref | *SERINC2* | Serine incorporator 2 |

Additional file 7 continued.

| ECA | Position (bp) | Genotype  Duelmener | Arabian | Sorraia | Hanoverian 1 | Hanoverian 2 | Gene  (human/ mouse orthologue) | Function |
| --- | --- | --- | --- | --- | --- | --- | --- | --- |
| Codon change plus codon insertion | | | |  |  |  |  |  |
| 2 | g.46251347ins | ref/ref | del/ref | ref/ref | ref/ref | ref/ref | *C1orf174* | Chromosome 1 open reading frame 174 |
| 2 | g.46251370ins | ref/ref | del/ref | ref/ref | ref/ref | ref/ref | *MLL5* | lysine (K)-specific methyltransferase 2E |
| 4 | g.5508562ins | ref/ref | del/ref | ref/ref | ref/ref | ref/ref | *SRPK2* | SRSF protein kinase 2 |
| 18 | g.55571695ins | ref/ref | del/ref | ref/ref | ref/ref | ref/ref | *HNRNPA3* | Heterogeneous nuclear ribonucleoprotein A3 |
| 18 | g.55571699ins | ref/ref | del/ref | ref/ref | ref/ref | ref/ref | *SERINC2* | Serine incorporator 2 |
| Codon deletion | | |  |  |  |  |  |  |
| 1 | g.15622864del | ref/ref | del/ref | ref/ref | ref/ref | ref/ref | *PNLIPRP3* | Pancreatic lipase-related protein 3 |
| 1 | g.60431877del | ref/ref | del/del | ref/ref | ref/ref | ref/ref | *MICU1* | Mitochondrial calcium uptake 1 |
| 3 | g.10338126del | ref/ref | del/ref | ref/ref | ref/ref | ref/ref | *PRSS54* | Protease, serine, 54 |
| 6 | g.69875591del | ref/ref | del/del | ref/ref | ref/ref | ref/ref | *KRT3* | Keratin 3 |
| 7 | g.75017217del | ref/ref | del/ref | ref/ref | ref/ref | ref/ref | *ENSECAG00000005147 (OR56A4)* | olfactory receptor, family 56 |
| 9 | g.82558935del | ref/ref | del/del | ref/ref | ref/ref | ref/ref | *PARP10* | Poly (ADP-ribose) polymerase family, member 10 |
| 9 | g.82558941del | ref/ref | del/del | ref/ref | ref/ref | ref/ref | *PARP10* | Poly (ADP-ribose) polymerase family, member 10 |
| 11 | g.24600859del | ref/ref | del/ref | ref/ref | ref/ref | ref/ref | *HOXB3* | Homeobox B3 |
| 12 | g.685612del | ref/ref | del/del | ref/ref | ref/ref | ref/ref | *TCP11L1* | T-complex 11, testis-specific-like 1 |

Additional file 7 continued.

| ECA | Position (bp) | Genotype  Duelmener | Arabian | Sorraia | Hanoverian 1 | Hanoverian 2 | Gene  (human/ mouse orthologue) | Function |
| --- | --- | --- | --- | --- | --- | --- | --- | --- |
| Codon deletion | | |  |  |  |  |  |  |
| 12 | g.13206424del | ref/ref | del/ref | ref/ref | ref/ref | ref/ref | *OR5I1* | Olfactory receptor, family 5, subfamily I, member 1 |
| 14 | g.31507281del | ref/ref | del/ref | ref/ref | ref/ref | ref/ref | *RBM27* | RNA binding motif protein 27 |
| 15 | g.29535174del | ref/ref | del/ref | ref/ref | ref/ref | ref/ref | *ALMS1* | Alstrom syndrome 1 |
| 16 | g.86209230del | ref/ref | del/del | ref/ref | ref/ref | ref/ref | *GPR149* | G protein-coupled receptor 149 |
| 20 | g.35958714del | ref/ref | del/ref | ref/ref | ref/ref | ref/ref | *SLC26A8* | Solute carrier family 26, member 8 |
| 22 | g.25607845del | ref/ref | del/ref | ref/ref | ref/ref | ref/ref | *NCOA6* | Nuclear receptor coactivator 6 |
| 25 | g.26070553del | ref/ref | del/ref | ref/ref | ref/ref | ref/ref | *ENSECAG00000005425* | Novel gene |
| Codon insertion | | |  |  |  |  |  |  |
| 20 | 29094006ins | del/ref | ref/ref | ref/ref | ref/ref | ref/ref | *ENSECAG00000023595* | Novel gene, 7-51 putative nonclassical MHC class I antigen |
| Codon change plus codon deletion | | | |  |  |  |  |  |
| 1 | g.30336306del | ref/ref | ref/ref | del/del | ref/ref | ref/ref | *ENSECAG00000022193 (GZMH)* | Novel gene  granzyme H |
| 3 | g.18227773del | ref/ref | ref/ref | del/ref | ref/ref | ref/ref | *NRN1L* | neuritin 1-like |
| 4 | g.10718901del | ref/ref | ref/ref | del/ref | ref/ref | ref/ref | *POU6F2* | POU class 6 homeobox 2 |
| 5 | g.73782194del | ref/ref | ref/ref | del/del | ref/ref | ref/ref | *BARHL2* | BarH-like homeobox 2 |

Additional file 7 continued.

| ECA | Position (bp) | Genotype  Duelmener | Arabian | Sorraia | Hanoverian 1 | Hanoverian 2 | Gene  (human/ mouse orthologue) | Function |
| --- | --- | --- | --- | --- | --- | --- | --- | --- |
| Codon change plus codon deletion | | | |  |  |  |  |  |
| 7 | g.25381742del | ref/ref | ref/ref | del/ref | ref/ref | ref/ref | *CEP164* | centrosomal protein 164kDa |
| 19 | g.21394460del | ref/ref | ref/ref | del/ref | ref/ref | ref/ref | *ATP11B* | ATPase, class VI, type 11B |
| 20 | g.26460848del | ref/ref | ref/ref | del/ref | ref/ref | ref/ref | *ENSECAG00000002140 (Olfr1535)* | Novel gene; olfactory receptor 1535 |
| 20 | g.62348733del | ref/ref | ref/ref | del/ref | ref/ref | ref/ref | *SMAP1* | Small ArfGAP 1 |
| 23 | g.22810567del | ref/ref | ref/ref | del/ref | ref/ref | ref/ref | *KANK1* | KN motif and ankyrin repeat domains 1 |
| 26 | g.30213441del | ref/ref | ref/ref | del/del | ref/ref | ref/ref | *SON* | SON DNA binding protein |
| Codon change plus codon insertion | | | | |  |  |  |  |
| 2 | g.41191496ins | ref/ref | ref/ref | del/ref | ref/ref | ref/ref | *UBE4B* | Ubiquitination factor E4B |
| 2 | g.69178041ins | ref/ref | ref/ref | del/ref | ref/ref | ref/ref | *ENSECAG00000009035 (ZNF284)* | Novel gene, zinc finger protein 284 |
| 7 | g.4740648ins | ref/ref | ref/ref | del/del | ref/ref | ref/ref | *ENSECAG00000024532 (ARHGEF18)* | Novel gene, Rho/Rac guanine nucleotide exchange factor (GEF) 18 |
| 11 | g.32268557ins | ref/ref | ref/ref | del/ref | ref/ref | ref/ref | *VEZF1* | Vascular endothelial zinc finger 1 |
| 22 | g.26321359ins | ref/ref | ref/ref | del/del | ref/ref | ref/ref | *ENSECAG00000009856 (Fer1l4)* | Novel gene, fer-1-like 4 (C. elegans) |

Additional file 7 continued.

| ECA | Position (bp) | Genotype  Duelmener | Arabian | Sorraia | Hanoverian 1 | Hanoverian 2 | Gene  (human/ mouse orthologue) | Function |
| --- | --- | --- | --- | --- | --- | --- | --- | --- |
| Codon deletion | | |  |  |  |  |  |  |
| 3 | g.34341915del | ref/ref | ref/ref | del/ref | ref/ref | ref/ref | *KLHDC4* | Kelch domain containing 4 |
| 3 | g.64622443del | ref/ref | ref/ref | del/del | ref/ref | ref/ref | *CABS1* | Calcium-binding protein, spermatid-specific 1 |
| 8 | g.43204427del | ref/ref | ref/ref | del/ref | ref/ref | ref/ref | *EMILIN2* | Elastin microfibril interfacer 2 |
| 21 | g.2273359del | ref/ref | ref/ref | del/ref | ref/ref | ref/ref | *USHBP1* | Usher syndrome 1C binding protein 1 |
| 23 | g.13462985del | ref/ref | ref/ref | del/ref | ref/ref | ref/ref | *CEP78* | Centrosomal protein 78kDa |
| 28 | g.2032602del | ref/ref | ref/ref | del/del | ref/ref | ref/ref | *FGD6* | FYVE, RhoGEF, PH domain containing 6 |
| Codon insertion | | |  |  |  |  |  |  |
| 6 | g.1141676ins | ref/ref | ref/ref | del/ref | ref/ref | ref/ref | *LANCL1* | LanC lantibiotic synthetase component C-like 1 (bacterial) |
| 18 | g.9225808ins | ref/ref | ref/ref | del/del | ref/ref | ref/ref | *CLASP1* | Cytoplasmic linker associated protein 1 |
| 29 | g.14312175ins | ref/ref | ref/ref | del/del | ref/ref | ref/ref | *DNAJC1* | DnaJ (Hsp40) homolog, subfamily C, member 1 |
| Codon change plus codon deletion | | | |  |  |  |  |  |
| 14 | g. 35424498del | ref/ref | ref/ref | ref/ref | del/del | del/del | *DIAPH1* | Diaphanous-related formin 1 |

Additional file 7 continued.

| ECA | Position (bp) | Genotype  Duelmener | Arabian | Sorraia | Hanoverian 1 | Hanoverian 2 | Gene  (human/ mouse orthologue) | Function |
| --- | --- | --- | --- | --- | --- | --- | --- | --- |
| Codon change plus codon insertion | | | |  |  |  |  |  |
| 1 | g.30114121ins | ref/ref | ref/ref | ref/ref | del/del | del/del | *NKX2-3* | NK2 homeobox 3 |
| 1 | g.157157697ins | ref/ref | ref/ref | ref/ref | del/ref | del/del | *TEP1* | Telomerase-associated protein 1 |
| 1 | g.157157699ins | ref/ref | ref/ref | ref/ref | del/del | del/del | *TEP1* | Telomerase-associated protein 1 |
| 8 | g.641070ins | ref/ref | ref/ref | ref/ref | del/ref | del/del | *ENSECAG00000015213* | Novel gene |
| Codon deletion | | |  |  |  |  |  |  |
| 5 | g.72748490del | ref/ref | ref/ref | ref/ref | del/del | del/ref | *BRDT* | Bromodomain, testis-specific |
| 6 | g.69875554del | ref/ref | ref/ref | ref/ref | del/ref | del/ref | *KRT3* | Keratin 3 |
| 6 | g.69875555del | ref/ref | ref/ref | ref/ref | del/ref | del/ref | *HIPK3* | Homeodomain interacting protein kinase 3 |
| 12 | g.418307del | ref/ref | ref/ref | ref/ref | del/ref | del/ref | *ENSECAG00000009122* | Novel gene |
| 13 | g.4816964del | ref/ref | ref/ref | ref/ref | del/del | del/ref | *BRDT* | Bromodomain, testis-specific |
