# Supplementary material for: Next generation sequencing gives an insight into the characteristics of highly selected breeds versus non-breed horses in the course of domestication
Source: BMC Genomics. 2014 Jul 4;15(1):562. doi: 10.1186/1471-2164-15-562 (PMC4097168; doi:10.1186/1471-2164-15-562)
Supplement: Supplementary file 9 — Additional file 9: Enrichment analysis of significantly overrepresented genes involved in biological processes. The software PANTHER was used for the evaluation of SNPs in coding regions and regulative regions for non-breed and breed horses. Raw P-values and Bonferoni corrected significant P-values are shown. (DOCX 32 KB) [file 12864_2013_6235_MOESM9_ESM.docx]

Additional file 9. Enrichment analysis of significantly overrepresented genes involved in biological processes. The software PANTHER was used for the evaluation of SNPs in coding regions and regulative regions for non-breed and breed horses. Raw P-values and Bonferoni corrected significant P-values are shown.

|  | Number of reference genes (Homo sapiens) | Number of genes in SNP regions (Horse) | Expected | Raw P-value | Bonferroni corrected P-value for multiple testing | Expected | Raw P-value | Bonferroni corrected P-value for multiple testing |
| --- | --- | --- | --- | --- | --- | --- | --- | --- |
| **coding regions** |  |  | non-breed (Duelmener and Sorraia) | | | breed (Hanoverian, Arabian) | | |
| muscle contraction | 559 | 43 | ─ | ─ | ─ | 21.94 | 1.40E-04 | 2.39E-02 |
| metabolic process | 8127 | 464 | 410.82 | 3.96E-04 | 6.77E-02 | ─ | ─ | ─ |
| primary metabolic process | 7813 | 450 | 394.95 | 2.38E-04 | 4.08E-02 | ─ | ─ | ─ |
| cellular component morphogenesis | 956 | 72 | 48.33 | 6.43E-04 | 1.10E-01 | ─ | ─ | ─ |
| anatomical structure morphogenesis | 956 | 72 | 48.33 | 6.43E-04 | 1.10E-01 | ─ | ─ | ─ |
| cellular process | 6072 | 355 | 306.94 | 6.54E-04 | 1.12E-01 | 238.33 | 3.31E-06 | 5.67E-04 |
| cell communication | 4224 | 361 | 273.5 | 1.99E-02 | 8.61E-07 | 165.79 | 9.93E-05 | 1.70E-02 |
| signal transduction | 4019 | 340 | 260.23 | 2.05E-02 | 8.82E-06 | 157.75 | 2.81E-04 | 4.80E-02 |

Additional file 9 continued.

|  | Number of reference genes (Homo sapiens) | Number of genes in SNP regions (Horse) | Expected | Raw P-value | Bonferroni corrected P-value for multiple testing | Expected | Raw P-value | Bonferroni corrected P-value for multiple testing |
| --- | --- | --- | --- | --- | --- | --- | --- | --- |
| **coding regions** |  |  | non-breed (Duelmener and Sorraia) | | | breed (Hanoverian, Arabian) | | |
| lipid metabolic process | 1047 | 63 | ─ | ─ | ─ | 41.09 | 6.43E-04 | 1.10E-01 |
| system process | 2159 | 197 | ─ | ─ | ─ | 84.74 | 7.04E-05 | 1.20E-02 |
| transport | 2679 | 232 | ─ | ─ | ─ | 105.15 | 1.14E-05 | 1.96E-03 |
| cell-cell signaling | 1259 | 74 | ─ | ─ | ─ | 49.42 | 4.27E-04 | 7.30E-02 |
| system development | 1911 | 104 | ─ | ─ | ─ | 75.01 | 4.74E-04 | 8.11E-02 |
| neurological system process | 1917 | 171 | ─ | ─ | ─ | 75.24 | 1.56E-04 | 2.67E-02 |
| sensory perception | 722 | 49 | ─ | ─ | ─ | 28.34 | 2.00E-04 | 3.42E-02 |
| nervous system development | 1146 | 70 | ─ | ─ | ─ | 44.98 | 2.14E-04 | 3.66E-02 |
| cation transport | 618 | 44 | ─ | ─ | ─ | 24.26 | 1.54E-04 | 2.63E-02 |
| ectoderm development | 1347 | 84 | ─ | ─ | ─ | 52.87 | 2.42E-05 | 4.13E-03 |
| developmental process | 2840 | 151 | ─ | ─ | ─ | 111.47 | 6.32E-05 | 1.08E-02 |

Additional file 9 continued.

|  | Number of reference genes (Homo sapiens) | Number of genes in SNP regions (Horse) | Expected | Raw P-value | Bonferroni corrected P-value for multiple testing | Expected | Raw P-value | Bonferroni corrected P-value for multiple testing |
| --- | --- | --- | --- | --- | --- | --- | --- | --- |
| **coding regions** |  |  | non-breed (Duelmener and Sorraia) | | | breed (Hanoverian, Arabian) | | |
| ion transport | 731 | 57 | ─ | ─ | ─ | 28.69 | 1.21E-06 | 2.06E-04 |
| intracellular signaling cascade | 1492 | 87 | ─ | ─ | ─ | 58.56 | 1.71E-04 | 2.93E-02 |
| **regulative regions** |  |  | non-breed (Duelmener and Sorraia) | | | breed (Hanoverian, Arabian) | | |

| primary metabolic process | 7813 | 502 | 397.29 | 1.78E-11 | 3.05E-09 | 392.6 | 2.03E-06 | 3.47E-04 |
| --- | --- | --- | --- | --- | --- | --- | --- | --- |
| metabolic process | 8127 | 516 | 413.26 | 4.96E-11 | 8.48E-09 | 408.38 | 4.42E-07 | 7.55E-05 |
| protein metabolic process | 3178 | 217 | 161.6 | 2.95E-06 | 5.05E-04 | 159.69 | 5.60E-04 | 9.58E-02 |
| system process | 2159 | 155 | 109.79 | 8.53E-06 | 1.46E-03 | 108.49 | 7.18E-08 | 1.23E-05 |
| neurological system process | 1917 | 137 | 97.48 | 3.79E-05 | 6.49E-03 | 96.33 | 8.29E-08 | 1.42E-05 |
| cellular process | 6072 | 368 | 308.76 | 3.97E-05 | 6.80E-03 | 305.12 | 1.39E-07 | 2.38E-05 |
| lipid metabolic process | 1047 | 83 | 53.24 | 6.00E-05 | 1.03E-02 | 52.61 | 4.70E-06 | 8.04E-04 |
| sensory perception | 722 | 62 | 36.71 | 6.32E-05 | 1.08E-02 | 36.28 | 4.21E-04 | 7.20E-02 |

Additional file 9 continued.

|  | Number of reference genes (Homo sapiens) | Number of genes in SNP regions (Horse) | Expected | Raw P-value | Bonferroni corrected P-value for multiple testing | Expected | Raw P-value | Bonferroni corrected P-value for multiple testing |
| --- | --- | --- | --- | --- | --- | --- | --- | --- |
| **regulative regions** |  |  | non-breed (Duelmener and Sorraia) | | | breed (Hanoverian, Arabian) | | |

| proteolysis | 1131 | 88 | 57.51 | 6.82E-05 | 1.17E-02 | ─ | ─ | ─ |
| --- | --- | --- | --- | --- | --- | --- | --- | --- |
| fatty acid metabolic process | 227 | 26 | 11.54 | 1.56E-04 | 2.66E-02 | ─ | ─ | ─ |
| signal transduction | 4019 | 250 | 204.37 | 2.79E-04 | 4.77E-02 | 201.95 | 3.92E-06 | 6.71E-04 |
| system development | 1911 | 152 | ─ | ─ | ─ | 96.03 | 2.00E-04 | 3.42E-02 |
| nervous system development | 1146 | 96 | ─ | ─ | ─ | ─ | ─ | ─ |
| exocytosis | 332 | 38 | ─ | ─ | ─ | 16.68 | 4.17E-06 | 7.14E-04 |
| synaptic vesicle exocytosis | 137 | 18 | ─ | ─ | ─ | 6.88 | 2.81E-04 | 4.81E-02 |
| developmental process | 2840 | 194 | ─ | ─ | ─ | 142.71 | 3.40E-04 | 5.82E-02 |
| transport | 2679 | 183 | ─ | ─ | ─ | 134.62 | 3.22E-05 | 5.50E-03 |
| cell communication | 4224 | 268 | ─ | ─ | ─ | 212.26 | 1.07E-06 | 1.83E-04 |
| vesicle-mediated transport | 1061 | 84 | ─ | ─ | ─ | 53.32 | 3.77E-05 | 6.45E-03 |

Additional file 9 continued.

|  | Number of reference genes (Homo sapiens) | Number of genes in SNP regions (Horse) | Expected | Raw P-value | Bonferroni corrected P-value for multiple testing | Expected | Raw P-value | Bonferroni corrected P-value for multiple testing |
| --- | --- | --- | --- | --- | --- | --- | --- | --- |
| **regulative regions** |  |  | non-breed (Duelmener and Sorraia) | | | breed (Hanoverian, Arabian) | | |

| protein transport | 1542 | 113 | ─ | ─ | ─ | 77.49 | 4.53E-05 | 7.74E-03 |
| --- | --- | --- | --- | --- | --- | --- | --- | --- |
| intracellular protein transport | 1542 | 113 | ─ | ─ | ─ | 77.49 | 4.53E-05 | 7.74E-03 |
| sensory perception of sound | 123 | 16 | ─ | ─ | ─ | 6.18 | 6.63E-04 | 1.13E-01 |
